# Dietary palmitic acid inhibits colorectal cancer progression through enhancing bisecting GlcNAc

**DOI:** 10.1172/jci.insight.179533

**Published:** 2026-03-23

**Authors:** Lei Lei, Juan Tang, Yuejiao Lv, Bingyi Jia, Wenqing Cai, Shuangshuang Sheng, Keying Li, Zhiwen Shi, Ning Fan, Zengqi Tan, Xiang Li, Feng Guan

**Affiliations:** 1 Key Laboratory of Resource Biology and Biotechnology Western China, Ministry of Education, Provincial Key Laboratory of Biotechnology, College of Life Sciences, Northwest University, Xi’an, China.; 2 Institute of Hematology, School of Medicine, Northwest University, Xi’an, China.

**Keywords:** Gastroenterology, Oncology, Colorectal cancer

## Abstract

Glycosylation changes are pivotal in colorectal cancer (CRC) development. The role of bisecting GlcNAc, a specific N-glycosylation type catalyzed by glycosyltransferase MGAT3, in CRC progression remains elusive. Previous studies indicated that dietary interventions can be beneficial for patients with certain congenital disorders of glycosylation. However, the impact of dietary fatty acids, such as palmitic acid (PA), on glycosylation regulation remains largely unclear. Here, we observed markedly decreased levels of bisecting GlcNAc and MGAT3 in colonic tissues of CRC patients. Downregulation of bisecting GlcNAc in CRC cells increased cell proliferation, migration, and invasion, while decreasing apoptosis. Moreover, a PA-rich diet inhibited CRC carcinogenesis in azoxymethane/dextran sodium sulfate–induced CRC mice by elevating bisecting GlcNAc levels. However, in Mgat3*^fl/fl^* Villin-Cre mice the inhibitory effects of the PA-rich diet were abolished. Intact glycopeptide analysis revealed that PA enhanced the bisecting GlcNAc modification on desmoglein 2 (DSG2). Additionally, DSG2 was identified to inhibit CRC carcinogenesis through the EGFR/AKT signaling pathway. In conclusion, dietary PA suppresses CRC carcinogenesis by regulating bisecting GlcNAc modification on DSG2, providing a direct mechanistic link between dietary fatty acids and CRC.

## Abstract 2

<a>Dietary palmitic acid suppresses CRC progression by enhancing bisecting GlcNAc modification on desmoglein 2,</a> providing a direct mechanistic link between dietary fatty acids and CRC.<a></a>

## Graphical abstract

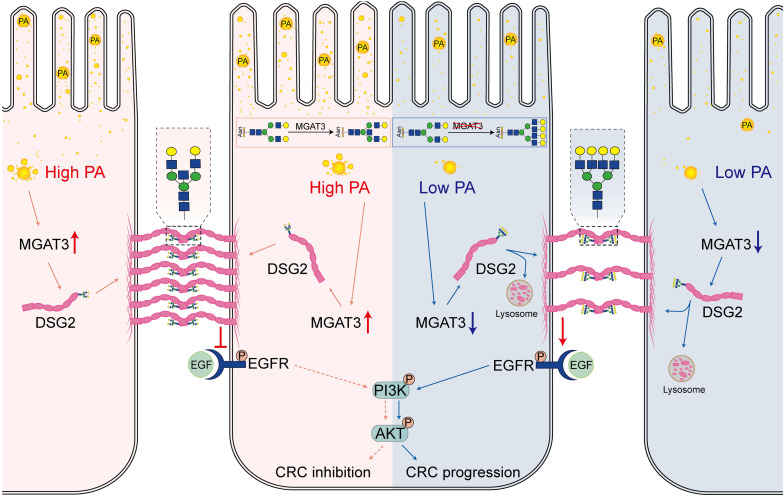

## Introduction

Colorectal cancer (CRC) is the third most prevalent malignant tumor worldwide (1). Early detection is pivotal, with the 5-year survival rate exceeding 90% for those diagnosed at stage I, compared with only 14% for patients with metastatic CRC (2). The process of carcinogenesis involves cancer cells acquiring various oncogenic features, including loss of cell adhesion and increased migratory and invasive capacities, ultimately leading to metastasis. These acquired biological characteristics of cancer cells depend on the genetic and epigenetic activation of key proteins regulating the migration process (3). Glycosylation is the most abundant and diverse form of posttranslational modification of proteins, including N-glycosylation, O-glycosylation, C-mannosylation, and GPI-anchored proteins (4). Notably, more than 50% of current cancer biomarkers are glycoproteins, which is unsurprising considering that glycosylation changes have emerged as crucial factors in cancer development (5).

Bisecting GlcNAc, a specific type of N-glycosylation involving the attachment of β1,4-linked GlcNAc to the core β-mannose residue, plays a role in suppressing the biosynthesis of terminal epitopes in N-glycans on proteins (6). Multiple glycomics studies suggest that bisecting GlcNAc could serve as a specific tumor marker for distinguishing between early and advanced stages of CRC (7, 8). Furthermore, a recent study has revealed that bisecting GlcNAc regulates IFN-γ receptor α protein stability and function in CRC cells (9). However, the precise molecular mechanisms by which bisecting GlcNAc regulates CRC development remain elusive.

Multiple studies indicated that specific glycosylation could act as a sensor of cellular nutritional state (10), and nutrition management through diet can be useful in certain patients with congenital disorders of glycosylation (11). Palmitic acid (PA), one of the most abundant saturated fatty acids (SFAs) in the human diet, functions as a signaling molecule involved in the regulation of various diseases, including CRC (12–14). Marth et al. demonstrated that PA treatment suppressed GnT-4a glycosyltransferase in pancreatic β cells (15). In this study, we found that PA increased the levels of bisecting GlcNAc in CRC cells, whereas monounsaturated fatty acids (MUFAs) and polyunsaturated fatty acids (PUFAs) had no such effect. Furthermore, a PA-rich diet inhibited CRC carcinogenesis and elevated the levels of bisecting GlcNAc in the azoxymethane/dextran sodium sulfate–induced (AOM/DSS-induced) CRC mouse model. This observation led us to hypothesize that PA inhibits CRC carcinogenesis through the modulation of bisecting GlcNAc. Our findings reveal intricate connections between dietary components, glycosylation dynamics, and CRC pathogenesis, potentially identifying innovative strategies for CRC prevention and treatment.

## Results

### Bisecting GlcNAc levels are reduced in CRC tissues.

**Figure 1:**
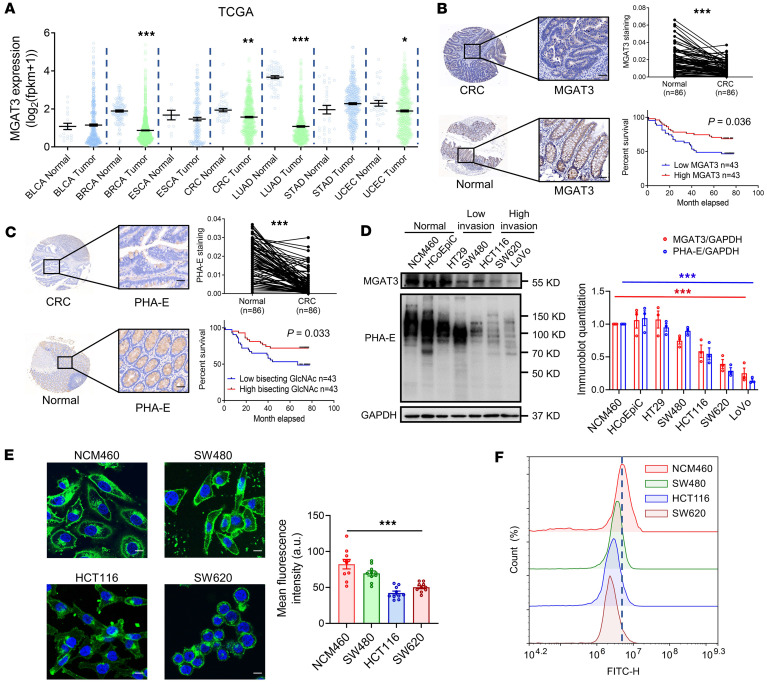
Bisecting GlcNAc levels are reduced in CRC tissues. (**A**) MGAT3 expression in TCGA database. BLCA, bladder cancer; BRCA, breast cancer; ESCA, esophageal cancer; LUAD, lung adenocarcinoma; STAD, stomach adenocarcinoma; UCEC, uterine corpus endometrial carcinoma. (**B**) Immunohistochemical (IHC) staining of MGAT3 in CRC and matched adjacent tissues on TMAs (scale bars: 50 μm), with corresponding Kaplan-Meier survival curves stratified by MGAT3 levels. (**C**) IHC staining of bisecting GlcNAc in CRC and matched adjacent tissues on TMAs (scale bars: 50 μm), with corresponding Kaplan-Meier survival curves stratified by bisecting GlcNAc levels. Bisecting GlcNAc levels in normal colon cells and CRC cells with low or high metastatic potential were evaluated by lectin blotting (**D**), immunofluorescence with *Phaseolus* *vulgaris* erythroagglutinating (PHA-E) lectin staining (see Supplemental Methods) (**E**, scale bars: 10 μm), and flow cytometry (**F**). The results are presented as mean ± SEM. The statistical significance of 2 groups was determined using a paired *t* test (**B** and **C**) or 2-tailed Student’s *t* test (**A**). Analysis of multiple groups was performed by 1-way ANOVA followed by Tukey’s multiple comparisons test (**D** and **E**). The cell culture experiments were performed with at least 3 independent repeats. *P < 0.05; **P < 0.01; ***P < 0.001.

We previously reported the downregulation of MGAT3 expression and bisecting GlcNAc levels in breast cancer (16, 17). To further investigate its potential role in other cancer types, we analyzed The Cancer Genome Atlas (TCGA) database and found a consistent reduction in MGAT3 expression in CRC, lung adenocarcinoma, and endometrioid cancer tissues compared with normal tissues (Figure 1A). Immunohistochemical (IHC) staining of CRC tissue microarrays confirmed a marked decrease in both MGAT3 expression and bisecting GlcNAc levels in CRC tissue compared with normal tissue (Figure 1, B and C). Notably, higher MGAT3 expression and elevated bisecting GlcNAc levels were positively correlated with improved overall survival in patients with CRC (Figure 1, B and C).

Quantitative analysis of Western blot data from 3 independent experiments confirmed that MGAT3 protein levels were markedly reduced in CRC cells relative to normal cells, consistent with the observed decrease in bisecting GlcNAc (Figure 1D). Highly metastatic CRC cells exhibited a downregulation of bisecting GlcNAc compared with cells with lower metastatic potential (Figure 1D). However, global N-glycosylation levels were not altered in CRC cells with different migration abilities (Supplemental Figure 1; supplemental material available online with this article; https://doi.org/10.1172/jci.insight.179533DS1). These findings were further substantiated through immunofluorescence and flow cytometry experiments (Figure 1, E and F). Bisecting GlcNAc was predominantly localized to the cell membrane, with a subset detected intracellularly, consistent with its role in modifying membrane and secretory proteins (6, 18). Quantification of fluorescence intensity confirmed a substantial reduction in both total and plasma membrane–associated bisecting GlcNAc levels in CRC cells, particularly in highly metastatic lines (HCT116 and SW620), relative to normal colon cells (NCM460) (Figure 1E).

### Bisecting GlcNAc levels are reduced in the colonic tissue of CRC mice.

**Figure 2:**
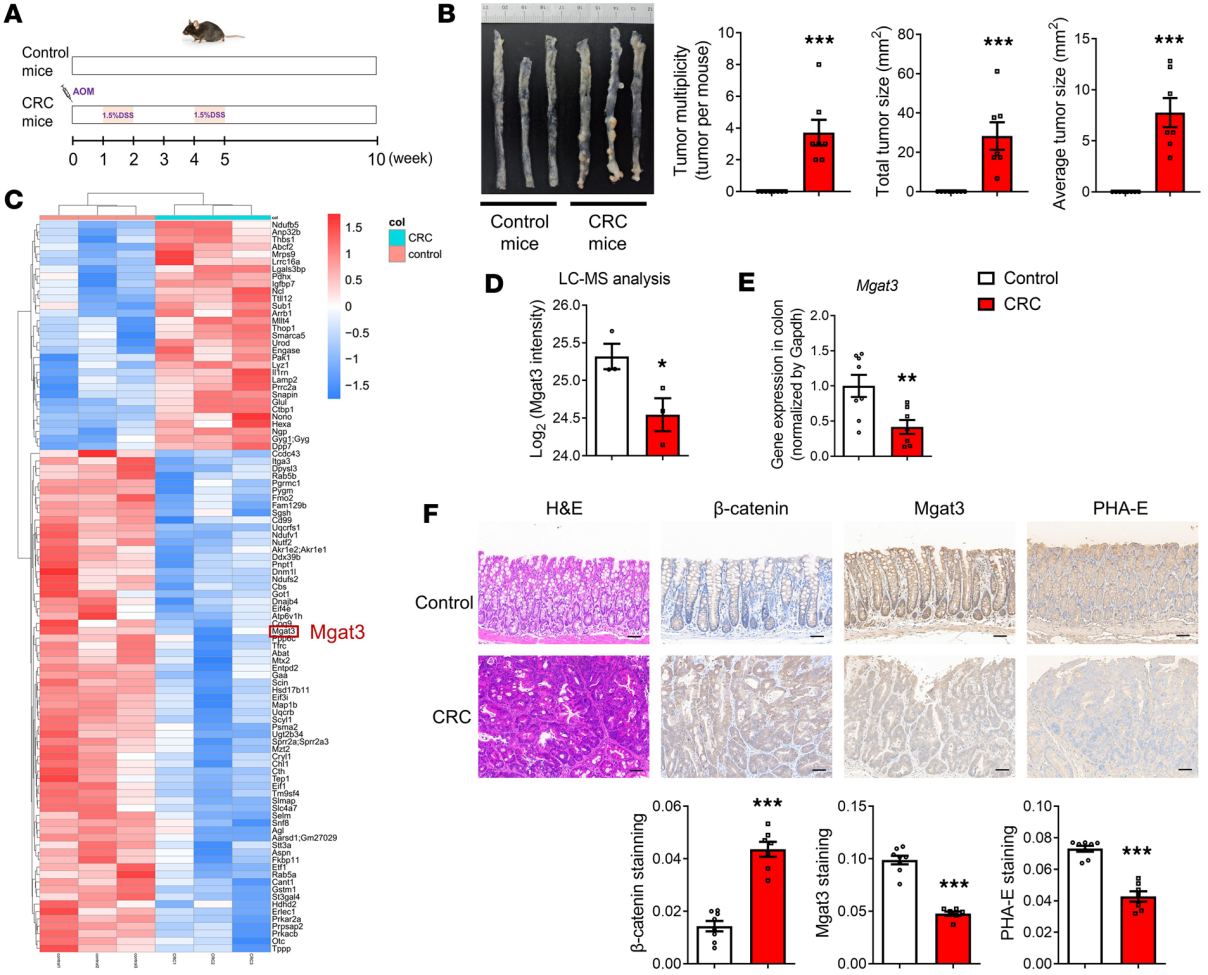
Bisecting GlcNAc levels are reduced in the colonic tissue of CRC mice. (**A**) Scheme of the animal experimental design. (**B**) Quantification of colon tumors in mice (*n* = 7–8 mice per group). (**C**) Heatmap of differentially expressed proteins in colonic tissues form CRC and control mice (*n* = 3 mice per group). (**D**) Mgat3 protein expression in colonic tissues form CRC and control mice. (**E**) *Mgat3* mRNA expression detected by qRT-PCR (*n* = 7–8 mice per group). (**F**) H&E staining and IHC for Pcna, Mgat3, and bisecting GlcNAc in colonic tissues (*n* = 7–8 mice per group, scale bars: 50 μm). The results are presented as mean ± SEM. The statistical significance of 2 groups was determined using a 2-tailed Student’s *t* test. *P < 0.05, **P < 0.01, ***P < 0.001.

We further generated AOM/DSS-induced CRC mouse models to assess Mgat3 expression and bisecting GlcNAc levels in colonic tissues from control and CRC mice (Figure 2, A and B). Proteomic analysis identified 99 differentially expressed proteins in the colonic tissues of CRC mice, including 31 upregulated and 68 downregulated proteins (Figure 2C and Supplemental Tables 1 and 2). Mgat3 expression was reduced in the colonic tissues of CRC mice (Figure 2D), and this reduction at the mRNA level was further validated by qRT-PCR (Figure 2E). IHC staining likewise demonstrated reduced levels of Mgat3 and bisecting GlcNAc in the colonic tissues of CRC mice, accompanied by elevated β-catenin expression (Figure 2F). Collectively, these findings indicate that downregulation of bisecting GlcNAc may contribute to CRC pathogenesis.

### Bisecting GlcNAc inhibits tumorigenesis of CRC cells.

**Figure 3:**
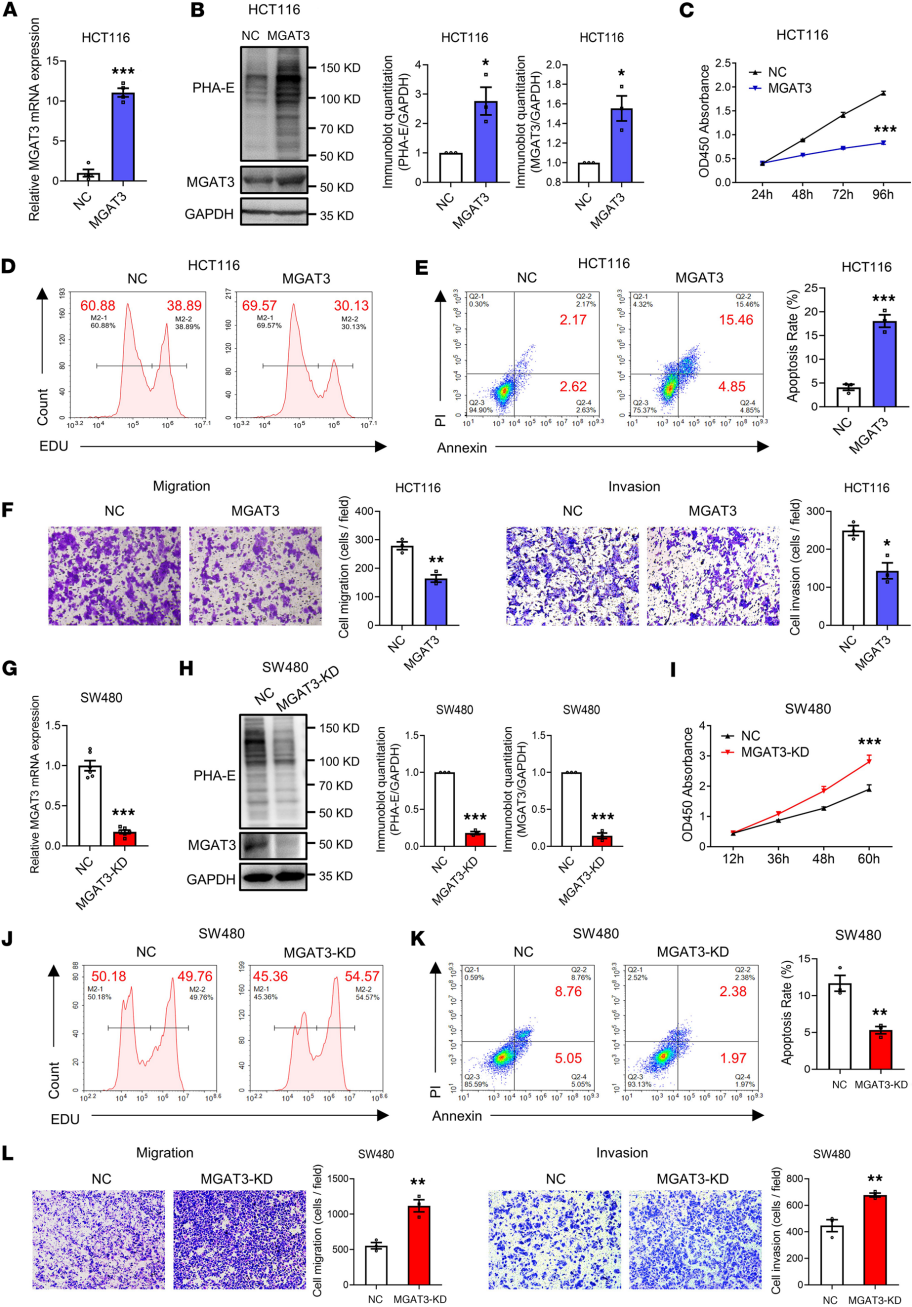
Bisecting GlcNAc inhibits tumorigenesis of CRC cells. MGAT3 overexpression in HCT116 cells was confirmed by qRT-PCR (**A**) and Western blotting (**B**). Cell proliferation of MGAT3-overexpressing HCT116 cells was assessed by CCK-8 (**C**) and EdU assays (**D**). (**E**) Apoptosis in MGAT3-overexpressing HCT116 cells. (**F**) Migratory and invasive capacities of MGAT3-overexpressing HCT116 cells. MGAT3 KD in SW480 cells was assessed by qRT-PCR (**G**) and Western blotting (**H**). Cell proliferation of MGAT3-KD SW480 cells was assessed by CCK-8 (**I**) and EdU assays (**J**). (**K**) Apoptosis in MGAT3-KD SW480 cells. (**L**) Migratory and invasive capacities of MGAT3-KD SW480 cells. The results are presented as mean ± SEM. The statistical significance of 2 groups was determined using a 2-tailed Student’s *t* test. The cell culture experiments were performed with at least 3 independent repeats. *P < 0.05, **P < 0.01, ***P < 0.001.

To investigate the potential influence of bisecting GlcNAc on CRC cell behavior, we overexpressed MGAT3 in HCT116 cells (Figure 3, A and B). Elevated bisecting GlcNAc levels inhibited cell proliferation, migration, and invasion, while promoting apoptosis (Figure 3, C–F). We then generated MGAT3-knockdown (MGAT3-KD) SW480 cells using CRISPR/Cas9-mediated gene editing (Figure 3, G and H). In contrast with MGAT3 overexpression, MGAT3 KD markedly enhanced cell proliferation, migration, and invasion, while reducing apoptosis (Figure 3, I–L). These findings highlight the inhibitory role of elevated bisecting GlcNAc in CRC carcinogenesis.

### PA increases bisecting GlcNAc levels in vitro and in vivo.

**Figure 4:**
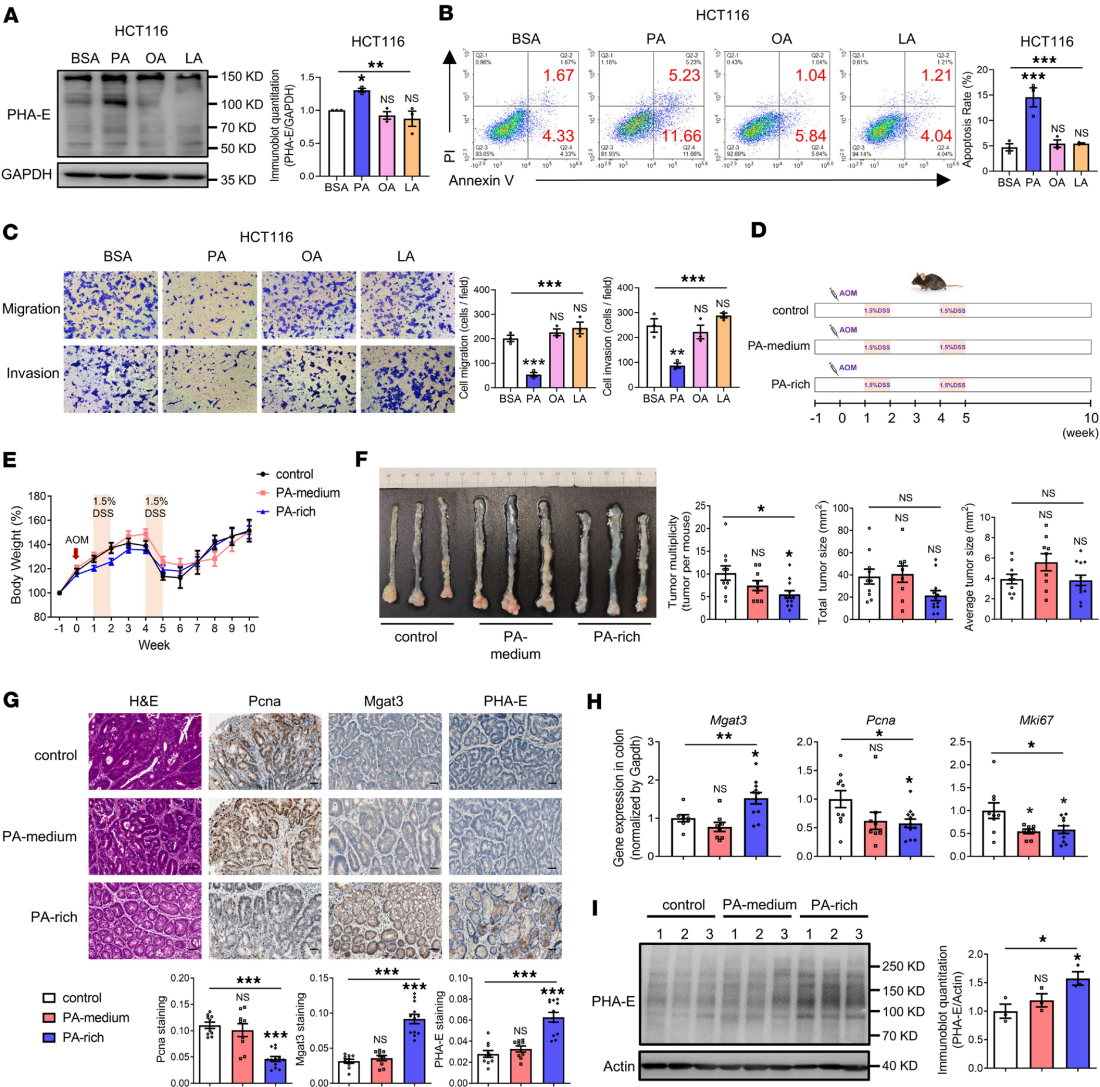
PA increases bisecting GlcNAc levels in vitro and in vivo. HCT116 cells were treated with PA, OA, LA, or BSA for 48 hours. (**A**) Bisecting GlcNAc levels assessed by lectin blotting. (**B**) Apoptosis detected by flow cytometry. (**C**) Migratory and invasive capacities assessed by Transwell assays. (**D**) Scheme of the animal experimental design. (**E**) Measurement of mouse body weight. (**F**) Quantification of colon tumors in mice (*n* = 9–12 mice per group). (**G**) H&E and IHC staining for Pcna, Mgat3, and bisecting GlcNAc in colonic tissues (*n* = 9–12 mice per group, scale bars: 50 μm). (**H**) mRNA expression of *Mgat3, Pcna*, and *Mki67* detected by qRT-PCR (*n* = 8–12 mice per group). (**I**) Bisecting GlcNAc levels in colonic tissues assessed by lectin blotting. The results are presented as mean ± SEM. Analysis of multiple groups was performed by 1-way ANOVA followed by Tukey’s multiple-comparison test. The cell culture experiments were performed with at least 3 independent repeats. NS, not significant. *P < 0.05; **P < 0.01; ***P < 0.001.

Previous studies indicated that PA downregulates Mgat4a expression, resulting in a deficiency of the GnT-4a glycosyltransferase in pancreatic β cells (15). To assess whether PA similarly affects bisecting GlcNAc levels*,* CRC cells were treated with different fatty acids: PA (a saturated fatty acid), linoleic acid (LA, a polyunsaturated fatty acid), and oleic acid (OA, a monounsaturated fatty acid). PA treatment markedly elevated bisecting GlcNAc levels in HCT116 cells, whereas OA and LA had no such effect (Figure 4A). The maximum increase was observed after 48 hours of PA treatment (Supplemental Figure 2A). Therefore, subsequent functional assays were performed using 200 μM PA for 48 hours. Flow cytometry and Transwell assays demonstrated that PA markedly promoted apoptosis and inhibited the migratory and invasive capacities of HCT116 cells, with effects exceeding those of OA or LA (Figure 4, B and C). Consistent results were obtained in SW480 cells (Supplemental Figure 2, B–D).

Consistent with in vitro findings, in vivo results demonstrated a substantial reduction in total tumor number of mice fed a PA-rich diet compared with the control group, whereas no significant changes occurred in the PA-medium group (Figure 4, D–F). Furthermore, a PA-rich diet substantially downregulated the expression of protumorigenic genes (*Pcna* and *Mki67*) in the colon (Figure 4, G and H), accompanied by upregulation of bisecting GlcNAc level and Mgat3 expression (Figure 4, G–I). No significant changes occurred in the PA-medium group. Collectively, these results demonstrate that PA specifically increases the levels of bisecting GlcNAc and attenuates CRC carcinogenesis both in vitro and in vivo.

### PA inhibits CRC carcinogenesis through the regulation of bisecting GlcNAc.

**Figure 5:**
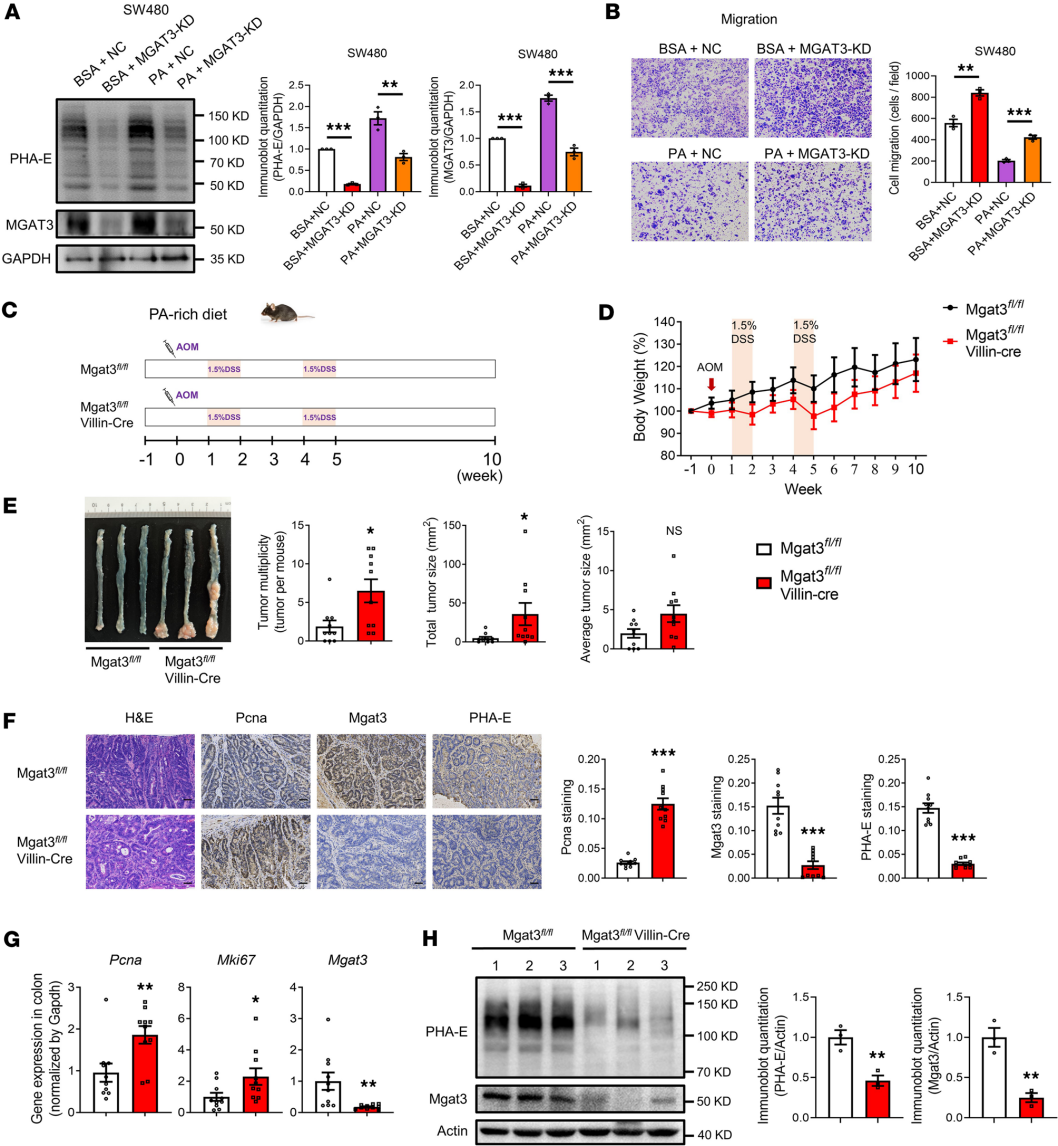
PA inhibits CRC carcinogenesis through the regulation of bisecting GlcNAc. (**A**) Bisecting GlcNAc levels and MGAT3 expression in MGAT3-KD SW480 cells treated with PA. (**B**) Migratory capacities assessed by Transwell assays. (**C**) Scheme of the animal experimental design. (**D**) Measurement of mouse body weight. (**E**) Quantification of colon tumors in mice (*n* = 10 mice per group). (**F**) H&E and IHC staining for Pcna, Mgat3, and bisecting GlcNAc in colonic tissues (*n* = 10 mice per group, scale bars: 50 μm). (**G**) mRNA expression of *Pcna, Mki67*, and *Mgat3* detected by qRT-PCR (*n* = 10 mice per group). (**H**) Mgat3 expression and bisecting GlcNAc levels in colonic tissues assessed by Western blotting and lectin blotting. The results are presented as mean ± SEM. The statistical significance of 2 groups was determined using a 2-tailed Student’s *t* test. The cell culture experiments were performed with at least 3 independent repeats. NS, not significant. *P < 0.05; **P < 0.01; ***P < 0.001.

We further treated MGAT3-KD SW480 cells with PA. Remarkably, MGAT3 KD (PA + MGAT3-KD) reversed the PA-induced (PA + negative control transfection [NC]) upregulation of MGAT3 and bisecting GlcNAc (Figure 5A). Moreover, MGAT3 KD (PA + MGAT3-KD) abolished the PA-induced (PA + NC) suppression of migration (Figure 5B). We generated Mgat3*^fl/fl^* Villin-Cre mice to further assess the impact of bisecting GlcNAc on the inhibitory effect of PA on CRC in vivo. First, both Mgat3*^fl/fl^* Villin-Cre mice and Mgat3*^fl/fl^* control mice were fed a normal diet and subjected to AOM/DSS to establish the CRC model. Mgat3*^fl/fl^* Villin-Cre mice on a normal diet developed markedly more tumors than Mgat3*^fl/fl^* control mice (Supplemental Figure 3), consistent with the tumor-suppressive role of Mgat3. Then, both Mgat3*^fl/fl^* Villin-Cre mice and Mgat3*^fl/fl^* mice were fed a PA-rich diet and subjected to the AOM/DSS treatment (Figure 5, C and D). Remarkably, compared with the control group (Mgat3*^fl/fl^*), the Mgat3*^fl/fl^* Villin-Cre group exhibited a marked increase in both total tumor number and size (Figure 5E). IHC, qRT-PCR, and Western blot analyses confirmed the KD of Mgat3 expression and bisecting GlcNAc levels in the colonic tissues of Mgat3*^fl/fl^* Villin-Cre mice (Figure 5, F–H). Additionally, upregulation of protumorigenic genes (*Pcna* and *Mki67*) occurred in the colonic tissues of Mgat3*^fl/fl^* Villin-Cre mice (Figure 5, F and G). Moreover, Mgat3*^fl/fl^* mice fed a PA-rich diet showed a marked reduction in tumor number compared with those fed a normal diet, with no significant difference in tumor number between Mgat3*^fl/fl^* Villin-Cre mice fed a PA-rich diet and those maintained on a normal diet (Supplemental Figure 3). These findings underscore the key role of bisecting GlcNAc in mediating the inhibitory effects of PA on CRC.

### PA stabilizes DSG2 expression through bisecting GlcNAc.

**Figure 6:**
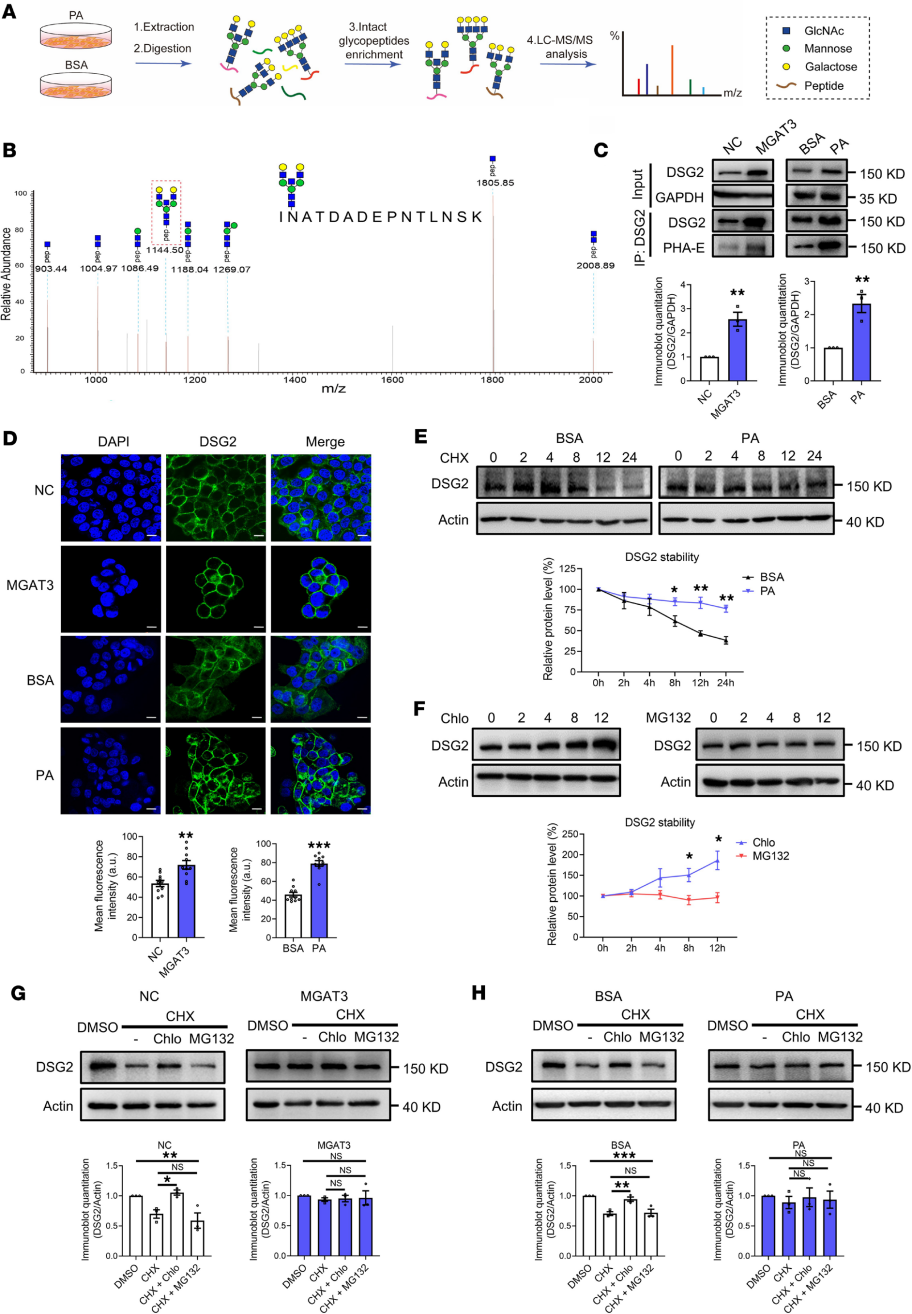
PA stabilizes DSG2 expression through bisecting GlcNAc. (**A**) Scheme of intact glycopeptide analysis. (**B**) Representative MS/MS spectrum of a DSG2-derived glycopeptide bearing bisecting GlcNAc. (**C**) Levels of bisecting GlcNAc–modified DSG2 in MGAT3-overexpressing or PA-treated HCT116 cells, detected by immunoprecipitation. (**D**) DSG2 immunofluorescence in MGAT3-overexpressing or PA-treated HCT116 cells (scale bars: 10 μm). (**E**) DSG2 half-life in BSA- or PA-treated HCT116 cells evaluated following cycloheximide (CHX) treatment. (**F**) DSG2 expression in HCT116 cells treated with chloroquine (Chlo) or MG132. DSG2 expression in MGAT3-overexpressing (**G**) or PA-treated (**H**) HCT116 cells following treatment with DMSO (8 hours), CHX (8 hours), CHX + Chlo (8 hours), or CHX + MG132 (8 hours). The results are presented mean ± SEM. The statistical significance of 2 groups was determined using a 2-tailed Student’s *t* test (**C**–**F**). Analysis of multiple groups was performed by 1-way ANOVA followed by Tukey’s multiple-comparison test (**G** and **H**). The cell culture experiments were performed with at least 3 independent repeats. NS, not significant. *P < 0.05; **P < 0.01; ***P < 0.001.

To identify specific glycoproteins regulated by PA-mediated bisecting GlcNAc modification, we performed intact glycopeptide analysis using liquid chromatography–tandem mass spectrometry (LC-MS/MS) (Figure 6A). Glycopeptide spectra were interpreted using Glyco-Decipher, identifying an average of over 200 glycopeptides and more than 130 glycoproteins per group (Supplemental Figure 4 and Supplemental Table 3). Characteristic ions diagnostic of bisecting GlcNAc–bearing glycopeptides ([pep+N3H] and [pep+N3HF]) (19) revealed that desmoglein 2 (DSG2) and lysosome-associated membrane protein 1 (LAMP1) were the only glycoproteins exhibiting this modification in PA-treated groups (Supplemental Table 3 and Figure 6B). Given DSG2’s established role in maintaining epithelial integrity (20), we focused on its functional relevance to bisecting GlcNAc–mediated effects. Immunoprecipitation assays confirmed the presence of bisecting GlcNAc on DSG2 and a marked increase in total DSG2 expression following both MGAT3 overexpression and PA treatment (Figure 6C). Furthermore, quantitative fluorescence imaging confirmed the enhanced DSG2 expression induced by these interventions (Figure 6D).

To investigate whether PA stabilizes DSG2 expression by inhibiting its degradation, we treated HCT116 cells with the protein synthesis inhibitor cycloheximide (CHX). PA treatment robustly prolonged the half-life of DSG2 (Figure 6E). Conversely, MGAT3 KD substantially reduced the half-life of DSG2 (Supplemental Figure 5). Additionally, the expression of DSG2 was enhanced by the lysosome pathway inhibitor chloroquine (Chlo) but was unaffected by the proteasome pathway inhibitor MG132 (Figure 6F). Similarly, treatment of HCT116 cells with CHX in combination with either Chlo or MG132 showed that the degradation of DSG2 in control samples (NC transfection and BSA treatment) was substantially reversed by Chlo, but not by MG132. In contrast, the degradation of DSG2 was similar after overexpression of MGAT3 or PA treatment (Figure 6, G and H). To investigate the subcellular sorting mechanism, we examined DSG2 localization relative to the lysosomal marker LAMP2, the early endosome marker EEA1, and the recycling endosome marker TFR (21). MGAT3 overexpression markedly decreased DSG2 colocalization with lysosomes, while increasing its colocalization with early endosomes and recycling endosomes (Supplemental Figure 6). Furthermore, both MGAT3 overexpression and PA treatment markedly reduced DSG2 accumulation in lysosome-enriched fractions (Supplemental Figure 7). Collectively, these findings indicate that PA stabilizes DSG2 expression by enhancing its bisecting GlcNAc modification, which promotes sorting into recycling endosomes and transport to the plasma membrane, thereby reducing lysosomal degradation of DSG2.

### PA inhibits CRC carcinogenesis through bisecting GlcNAc–modified DSG2.

**Figure 7:**
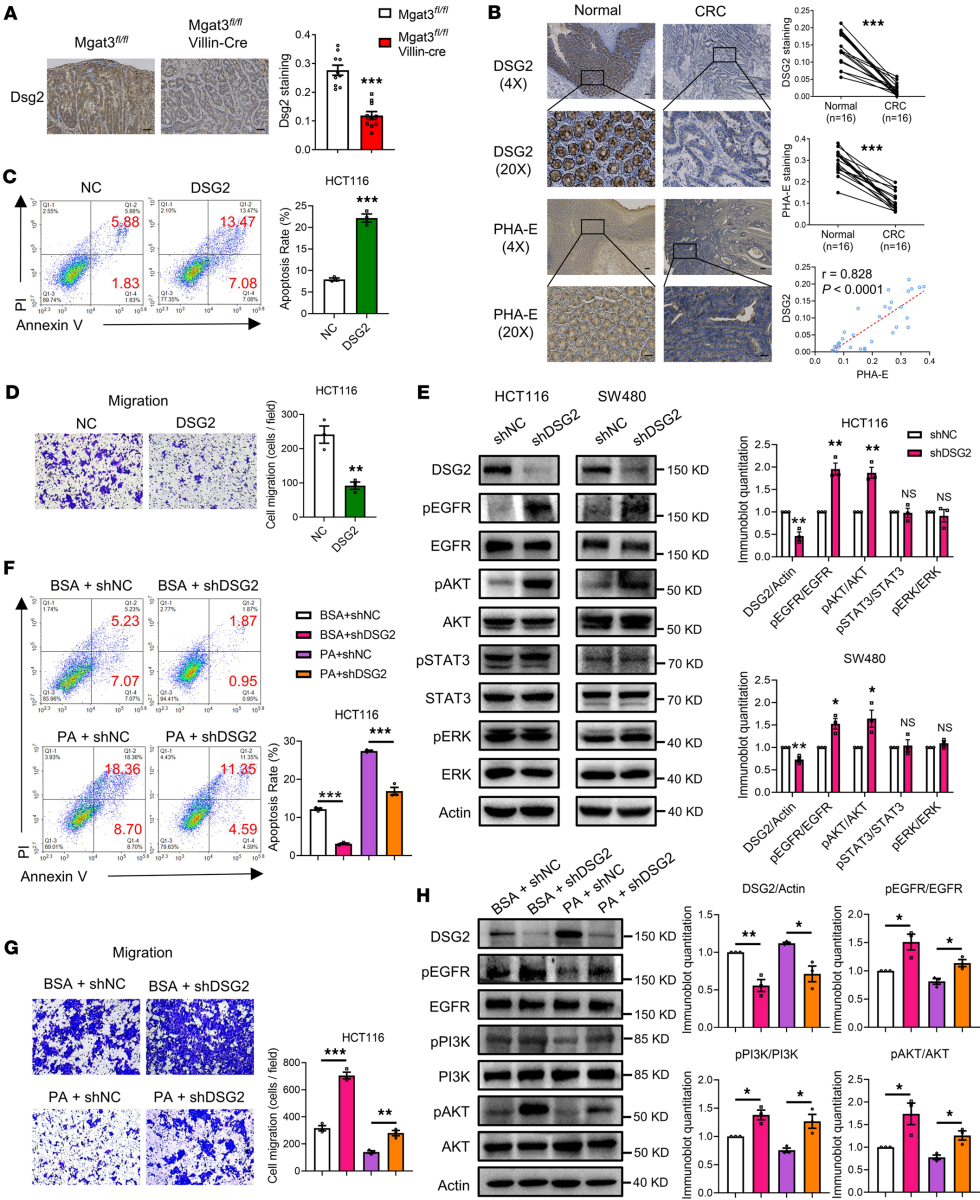
PA inhibits CRC carcinogenesis through bisecting GlcNAc–modified DSG2. (**A**) IHC staining of Dsg2 in mouse colonic tissues (*n* = 10 mice per group, scale bars: 50 μm). (**B**) IHC staining of DSG2 and bisecting GlcNAc in human CRC and adjacent normal tissues (*n* = 16 patients; 4× scale bars: 200 μm, 20× scale bars: 50 μm), with Pearson’s correlation analysis between DSG2 expression and bisecting GlcNAc levels. Apoptosis (**C**) and migratory abilities (**D**) of DSG2-overexpressing HCT116 cells. (**E**) Phosphorylation status of EGFR signaling pathway components in DSG2-KD HCT116 and SW480 cells. Apoptosis (**F**) and migratory abilities (**G**) of HCT116 cells treated with PA and shDSG2. (**H**) Phosphorylation status of EGFR signaling pathway in HCT116 cells treated with PA and shDSG2. The results are presented as mean ± SEM. The statistical significance of 2 groups was determined using a 2-tailed Student’s *t* test. The cell culture experiments were performed with at least 3 independent repeats. NS, not significant. *P < 0.05; **P < 0.01; ***P < 0.001.

Downregulation of Dsg2 was observed in the colonic tissues of Mgat3*^fl/fl^* Villin-Cre mice (Figure 7A). Furthermore, reduced expression of DSG2, which correlated with levels of bisecting GlcNAc, was observed in human CRC tissues (Figure 7B). Overexpression of DSG2 in HCT116 cells substantially increased apoptosis and decreased migration compared with the NC group (Figure 7, C and D). In contrast, downregulating DSG2 expression in both HCT116 and SW480 cells increased the phosphorylation of EGFR and AKT, but not STAT3 or ERK phosphorylation (Figure 7E). These results indicated that high DSG2 expression inhibits CRC carcinogenesis through the EGFR/AKT signaling pathway. We then examined the impact of PA on the DSG2 function in HCT116 cells and found that KD of DSG2 (PA + shDSG2) abolished the PA-induced (PA + shNC) enhancement of apoptosis (Figure 7F) and the suppression of migration (Figure 7G). Notably, PA treatment (PA + shNC) decreased the activation of the EGFR/AKT signaling pathway, which was reversed by DSG2 inhibition (PA + shDSG2) (Figure 7H). These findings confirm that PA inhibits CRC carcinogenesis through the regulation of DSG2 via bisecting GlcNAc.

## Discussion

CRC is a major global health burden, and understanding the impact of dietary factors on CRC development is crucial for prevention and treatment strategies. In this study, we investigated how dietary PA affects CRC progression through the regulation of bisecting GlcNAc, a specific form of N-glycosylation.

Bisecting GlcNAc suppresses various terminal modifications of N-glycans (6). Accumulating evidence indicates that dysregulation of bisecting GlcNAc is a critical event in CRC progression (7, 8). We previous reported that elevated bisecting GlcNAc reduces the prometastatic activity of small extracellular vesicles derived from breast cancer cells (17). Similarly, loss of bisecting GlcNAc on melanoma cell adhesion molecule (MCAM) in bone marrow stroma contributed to protumoral niche formation in myelodysplastic syndrome (22). Other studies have shown that downregulation of bisecting GlcNAc reduces the stability and function of IFN-γ receptor α protein, thereby promoting IFN-γ resistance in CRC cells (9). Despite these findings, the molecular mechanisms by which bisecting GlcNAc regulates CRC development remain unclear. In the present study, we observed a substantial reduction in bisecting GlcNAc and its glycosyltransferase MGAT3 in CRC tissues from both human patients and mouse models. A limitation of our survival analysis is that it relied on overall survival data from the tissue microarray. Future studies incorporating disease-specific survival information will enable more precise prognostic evaluation of bisecting GlcNAc and MGAT3. We further demonstrated that MGAT3 overexpression inhibited cell proliferation, migration, and invasion, while inducing apoptosis, whereas CRISPR/Cas9-mediated MGAT3 KD exerted the opposite effects. Together, these findings support an inhibitory role for bisecting GlcNAc in CRC carcinogenesis.

Lifestyle factors, particularly dietary habits, substantially influence the occurrence and progression of CRC (23). Current dietary guidelines recommend replacing SFAs with unsaturated fatty acids, such as MUFAs and PUFAs, for cardiovascular disease prevention (24–26). Although the cardioprotective effects of PUFAs remain debated (27), global PUFA consumption has increased substantially (28, 29). However, studies from our group and others suggest that high PUFA intake may elevate the risk of colonic inflammation and CRC (30–33). Conflicting findings have been reported regarding the impact of dietary PA on CRC. Some studies using a low-fat diet (LFD, 10 kcal% fat) as control showed that a PA-rich high-fat diet (HFD, 60 kcal% fat) promotes CRC growth in xenograft mouse models (14, 34). Conversely, studies using an *n*-6 PUFA–rich HFD (50.3 kcal% fat) as control found that a PA-rich diet (27.8 kcal% fat) reduced colonic inflammatory lesions and decreased expression of the inflammatory marker COX-2 (35). Additionally, compared with a lard-rich HFD (48 kcal% fat), a PA-rich HFD (48 kcal% fat) resulted in reduced white adipose tissue mass and improved glucose tolerance (36). These findings suggest that the tumor-promoting effects reported in earlier studies may be attributable to the high-fat diet itself rather than to PA specifically. Notably, we previously observed that mice fed an HFD exhibited reduced colonic PA levels compared with those on an LFD (37). In this study, using a modified AIN-93G diet (15.8 kcal% fat) in AOM/DSS-induced CRC mice, we found that a PA-rich diet markedly attenuated CRC tumorigenesis. Strikingly, this inhibitory effect was abolished in Mgat3*^fl/fl^* Villin-Cre mice, indicating that PA inhibits CRC carcinogenesis by modulating bisecting GlcNAc levels.

To elucidate the molecular mechanisms underlying dietary PA effects, we conducted intact glycopeptide analysis to systematically and unbiasedly identify target proteins regulated by PA-induced bisecting GlcNAc modification. Our results indicate that PA stabilizes DSG2 expression by enhancing its bisecting GlcNAc modification. DSG2, a cadherin-type adhesion molecule of desmosomes, is crucial for maintaining intestinal barrier integrity (20). In patients with Crohn disease, DSG2 protein levels are substantially reduced without corresponding mRNA changes, suggesting posttranslational regulation (38). Furthermore, DSG2-deficient mice exhibit enhanced intestinal epithelial barrier disruption and more severe colitis (39). However, the role of DSG2 in cancer development appears context dependent. Evidence supports a tumor-suppressive role for DSG2 in pancreatic and gallbladder cancers through inhibition of EGFR and its downstream signaling pathways (40, 41), whereas other studies have shown that DSG2 promotes lung cancer progression (42). In our study, elevated bisecting GlcNAc promoted DSG2 trafficking to recycling endosomes, facilitating its delivery to the plasma membrane and reducing lysosomal degradation. Increased DSG2 expression subsequently suppressed EGFR signaling, thereby mediating the inhibitory effect of PA on CRC development.

Regarding the regulation of MGAT3 expression, a previous study reported that the downregulation of β-catenin reduces MGAT3 expression (43). However, we found elevated β-catenin accompanied by reduced MGAT3 levels in CRC tissues (Figure 2F), suggesting that additional regulatory factors influence CRC. Furthermore, PA selectively increased bisecting GlcNAc without substantially altering global N-glycosylation (Supplemental Figure 8). Notably, MGAT3 promoter methylation is increased in CRC tissues compared with normal colonic tissues, which may contribute to its reduced expression in tumors. Previous studies have reported that PA can modulate cellular DNA methylation levels (44). Therefore, it is plausible that PA may influence MGAT3 expression through modulation of promoter methylation, an important direction for future investigation.

In summary, our findings demonstrate that dietary PA suppresses CRC carcinogenesis by modulating the bisecting GlcNAc modification of DSG2. These results provide a direct mechanistic link between dietary fatty acids and CRC progression, highlighting potential therapeutic targets for CRC prevention and treatment.

## Methods

Further information can be found in Supplemental Methods.

### Sex as a biological variable.

In human studies, both male and female patients were included in approximately equal numbers. In animal studies, male and female mice were used in this study. Sex was not considered as a biological variable.

### Human tissue.

Colonic tissue microarrays (TMAs) containing 86 cases of colon cancer and para-carcinoma tissues were obtained from Shanghai Outdo Biotech Co. Additionally, CRC and matched adjacent tissues from 16 patients were obtained from the Third Affiliated Hospital of Northwest University. Detailed immunostaining procedures are provided in the Supplemental Methods.

### CRISPR/Cas9-mediated MGAT3 KD.

MGAT3 KD in CRC cells was performed using the LentiCRISPR-Hygro system (Adgene). Briefly, guide RNAs targeting MGAT3 were designed and cloned into the LentiCRISPR-Hygro vector. Lentiviral particles were produced by cotransfecting HEK293T cells with the transfer plasmid, psPAX2, and pMD2.G using polyethyleneimine (PEI). After 48–72 hours, viral supernatants were collected, filtered, and used to infect SW480 or HCT116 cells with 8 μg/mL polybrene. Transduced cells were selected using hygromycin and KD efficiency was validated by Western blotting.

### Conventional and transgenic CRC mouse models.

For the conventional CRC model, C57BL/6 mice (Beijing Vital River Laboratory Animal Technology Co.) (6–7 weeks old) received intraperitoneal (i.p.) injections of 10 mg/kg AOM (Sigma-Aldrich). Subsequently, on weeks 1 and 4 after AOM injection, mice received 1.5% DSS (MP Biomedicals) in drinking water for 1 week. Mice were sacrificed for analysis on week 10 after AOM injection. Tumor multiplicity was assessed by counting the number of tumors per mouse, and tumor size was calculated using the following formula: tumor size = π × *d*^2^/4, where *d* is tumor diameter.

To assess the effects of dietary PA in the CRC model, C57BL/6 mice (6–7 weeks old) were randomly divided into 3 groups receiving diets with different PA content (Supplemental Table 4). Following 1 week of dietary adaptation, mice underwent AOM and DSS treatment. At week 10 after AOM injection, mice were sacrificed for colon tumor analysis.

For the transgenic CRC model, C57BL/6 Mgat3*^fl/fl^* Villin-Cre mice were generated through the crossbreeding of Mgat3*^fl/fl^* and B6.Cg-Tg(Vil1-cre)1000Gum/J mice (Villin-Cre). Mgat3*^fl/fl^* and B6.Cg-Tg(Vil1-cre)1000Gum/J mice were obtained from The Jackson Laboratory. Both Mgat3*^fl/fl^* Villin-Cre and Mgat3*^fl/fl^* mice (6–7 weeks old) were fed either a normal diet or a PA-rich diet throughout the experiment. Mice were sacrificed on week 10 after AOM treatment.

### Proteomic analysis.

Following sacrifice, entire colonic tissues (including tumor and adjacent normal areas) were collected. Approximately 50 mg tissue was homogenized in 500 μL lysis buffer (8 M urea,1 M NH_4_HCO_3_) and sonicated on ice. The supernatant was collected after centrifugation at 15,900*g* for 10 minutes. Proteins (100 μg) were reduced using 5 mM dithiothreitol (DTT), alkylated with 20 mM iodoacetamide (IAM), digested with lysyl endopeptidase (Wako Puro Chemical) for 4 hours at 37°C, and then digested with trypsin (Promega) overnight at 37°C. The mixture was acidified to a pH of less than 3 with 10% trifluoroacetic acid (TFA) and purified using Oasis HLB cartridges (Waters). LC-MS/MS analysis was performed using an ultra-high-performance LC (UHPLC) system coupled to a Q Exactive HFX mass spectrometer (Thermo Fisher Scientific). The Q Exactive HFX mass spectrometer was used to acquire MS/MS spectra in information-dependent acquisition mode in the control of the acquisition software (Xcalibur, Thermo Fisher Scientific). Quantification was performed using the MaxQuant software program, as described previously (45).

### Intact glycopeptide analysis.

HCT116 cells were treated with 200 μM PA or BSA for 48 hours. Subsequently, proteins (1 mg) were denatured in 8 M urea, 5 mM DTT, 20 mM IAM, and then digested with lysyl endopeptidase and trypsin. Peptides were purified using Oasis HLB cartridges, lyophilized, and resuspend in 200 μL 50% acetonitrile with 1% TFA. Glycopeptide enrichment was performed using Oasis MAX columns (Waters). LC-MS/MS and data analysis was carried out using the Q Exactive HFX mass spectrometer and Glyco-Decipher software (46). Proteins with bisecting N-glycans were identified as described previously (17).

### Statistics.

The expression data of MGAT3 in CRC were obtained from the UCSC Xena dataset (https://xena.ucsc.edu/). All data are presented as mean ± SEM and were analyzed using Prism 9 (GraphPad Software Inc.). Statistical comparisons employed Kaplan-Meier survival analysis, 1-way ANOVA, paired *t* tests, and 2-tailed Student’s *t* tests. Analysis of multiple groups was performed by 1-way ANOVA followed by Tukey’s multiple-comparison test. Correlations were assessed using Pearson’s correlation test. Statistical significance was established at *P* less than 0.05.

### Study approval.

The utilization of human tissues in experiments was approved by the Research Ethics Committee of Northwest University, Shaanxi, China. Written informed consent was obtained from all patients in accordance with the Declaration of Helsinki. All animal experiments complied with the Guide for the Care and Use of Laboratory Animals of the State Committee of Science and Technology of the People’s Republic of China. The protocols employed in this study were approved by the Institutional Animal Care and Use Committee of Northwest University, Shaanxi, China.

### Data availability.

All data in the article are included in the Supporting Data Values file and Supplemental Tables.

## Author contribution

LL designed and performed experiments, analyzed the data, and drafted the manuscript. JT, YL, and BJ performed mouse experiments. WC performed bioinformatics analyses. SS and KL performed cell experiments. ZS and NF performed histopathological evaluation. ZT and XL commented on the study, and revised the manuscript. FG supervised the study, provided the facilities, and revised the manuscript. All authors read and approved the final manuscript.

## Funding support

- National Natural Science Foundation of China grant nos. 32471333, 82172828, 32071274, and 32271338.
- Science Foundation for Distinguished Young Scholars of Shaanxi Province (2021JC-39).
- Natural Science Foundation of Shaanxi Province (2022JM-522, 2022JQ-205).
- Shaanxi Innovation Team Project (2023-CX-TD-58).

## Supplementary Material

Supplemental data

Unedited blot and gel images

Supplemental table 2

Supplemental table 3

Supporting data values

## Version 1 03/23/2026

Electronic publication
