# Supplementary material for: Dietary palmitic acid inhibits colorectal cancer progression through enhancing bisecting GlcNAc
Source: JCI Insight. 2026 Mar 23;11(6):e179533. doi: 10.1172/jci.insight.179533 (PMC13043105; doi:10.1172/jci.insight.179533)
Supplement: Supplemental data [file jciinsight-11-179533-s008.pdf]

Supplemental Text and Figures

Dietary palmitic acid inhibits colorectal cancer progression through enhancing bisecting GlcNAc

Lei Lei[1], Juan Tang[1], Yuejiao Lv[1], Bingyi Jia[1], Wenqing Cai[1], Shuangshuang Sheng[1], Keying Li[1], Zhiwen Shi[1], Ning Fan[1], Zenqqi Tan[1], Xiang Li[2], Feng Guan[1,2*]

[1]Key Laboratory of Resource Biology and Biotechnology Western China, Ministry of Education; Provincial Key Laboratory of Biotechnology, College of Life Sciences, Northwest University, Xi'an, 710069, China.

[2]Institute of Hematology, School of Medicine, Northwest University, Xi’an, 710069, China

***Correspondence**: Feng Guan, College of Life Sciences, Northwest University, No.229, Taibai North Road, Xi’an, Shaanxi 710069, China, E-mail: guanfeng@nwu.edu.cn, Phone: +86-29-88303534

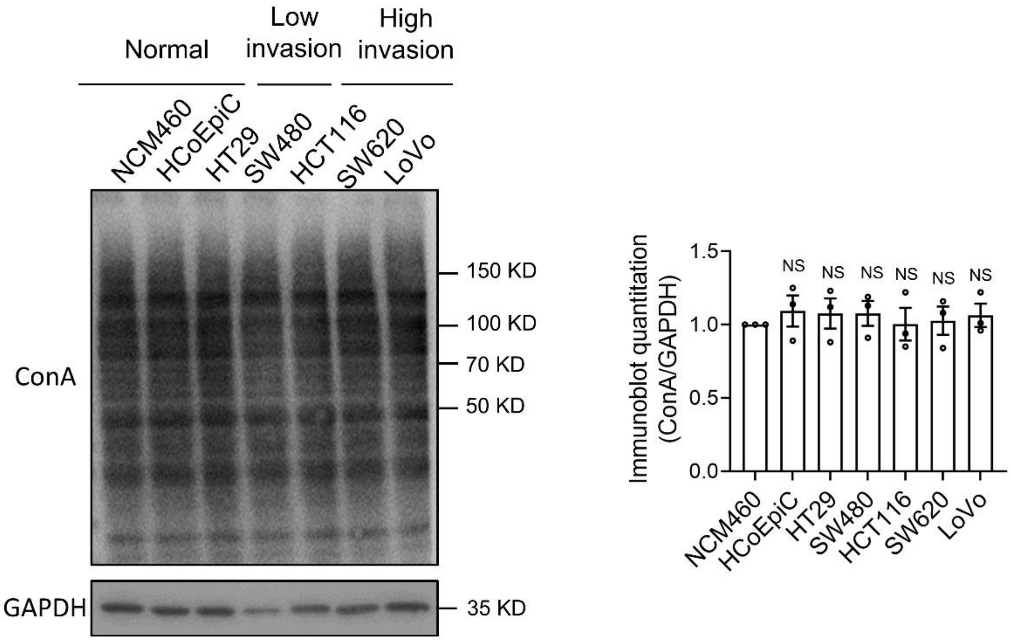

Figure S1. Global N-glycosylation levels were not altered in CRC cells with different migration abilities. Global N-glycosylation assessed by lectin blot with ConA (left). Quantification of three independent experiments (right).

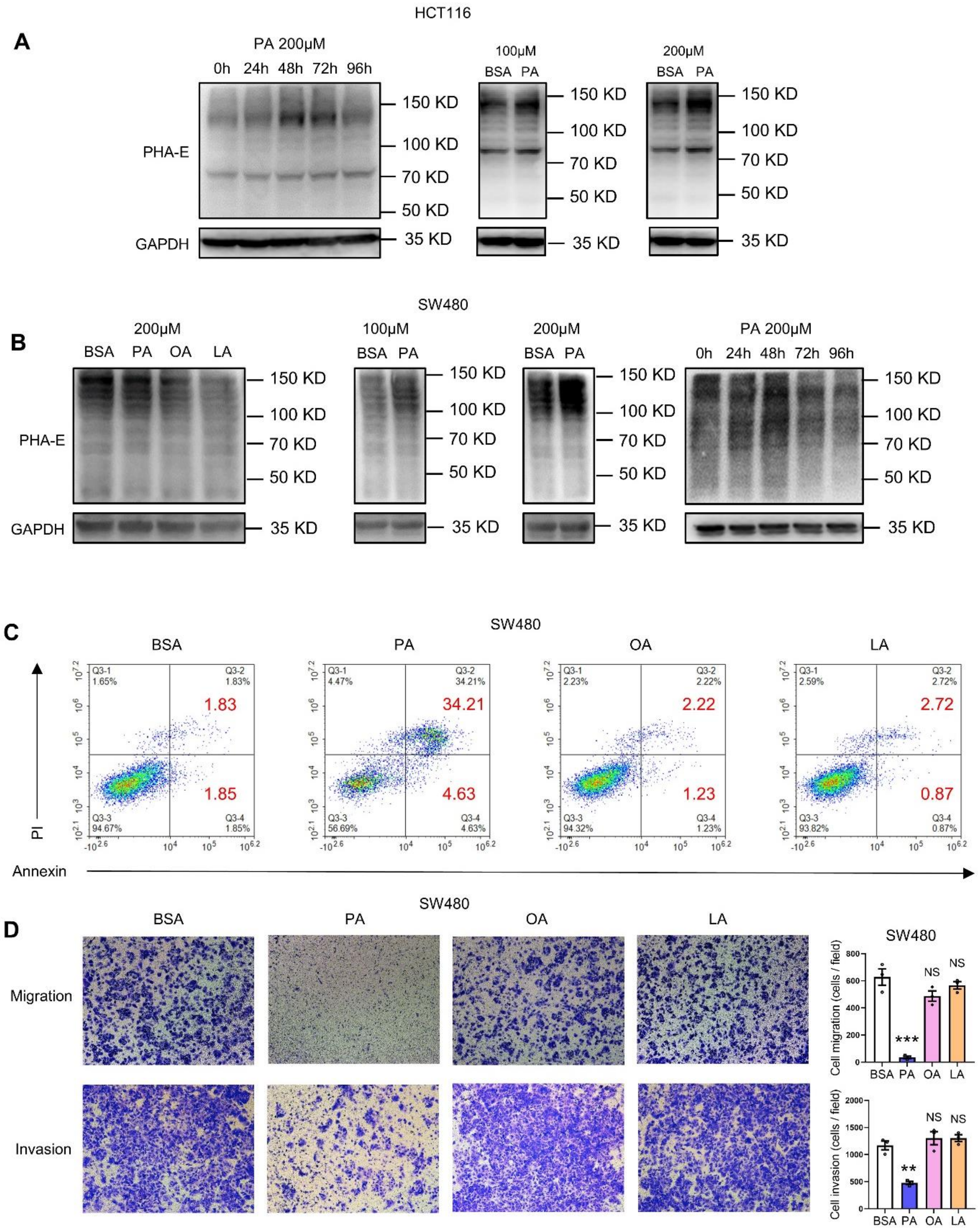

Figure S2. PA increased bisecting GlcNAc levels in HCT116 and SW480 cells. (**A and B**) Bisecting GlcNAc levels in HCT116 and SW480 cells treated with PA, OA, LA and BSA assessed by lectin blot. (**C**) Apoptosis of SW480 cells treated with PA, OA, LA and BSA detected by flow cytometry. (**D**) Migratory and invasive abilities of SW480 cells treated with PA, OA, LA and BSA detected by transwell assays.

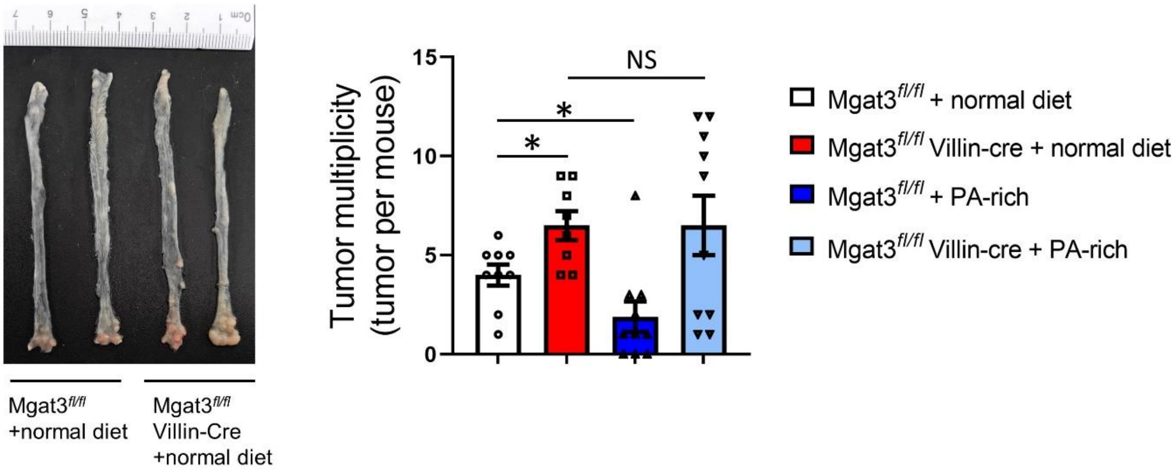

Figure S3. Mgat3$^{fl/fl}$ Villin-Cre mice on a normal diet developed more tumors than Mgat3$^{fl/fl}$ control mice. Image and quantification of colon tumors in mice (n = 8-9 mice per group)

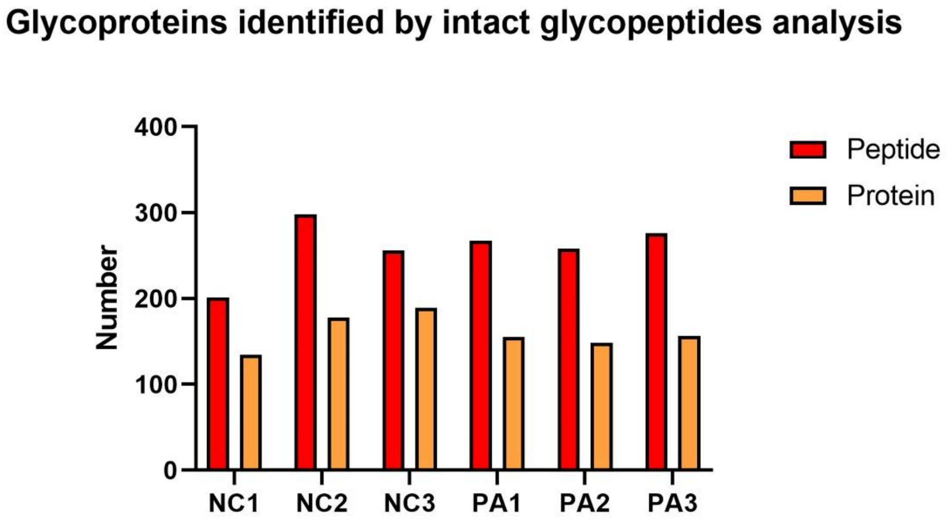

Figure S4. The number of glycopeptides and glycoproteins identified per group using Glyco-Decipher

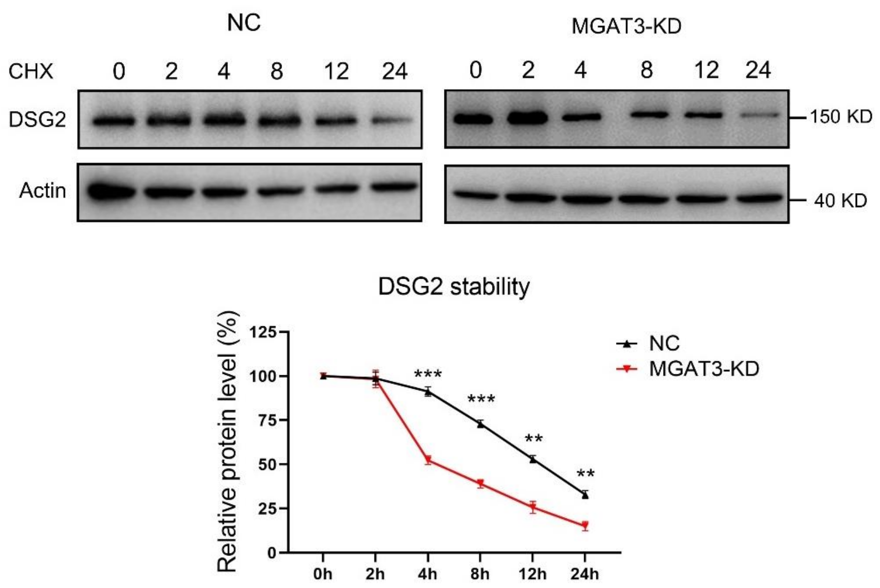

Figure S5. MGAT3 knockdown reduced the half-life of DSG2. DSG2 half-life in control and MGAT3-knockdown HCT116 cells evaluated following cycloheximide (CHX) treatment (up). Quantification of three independent experiments (down).

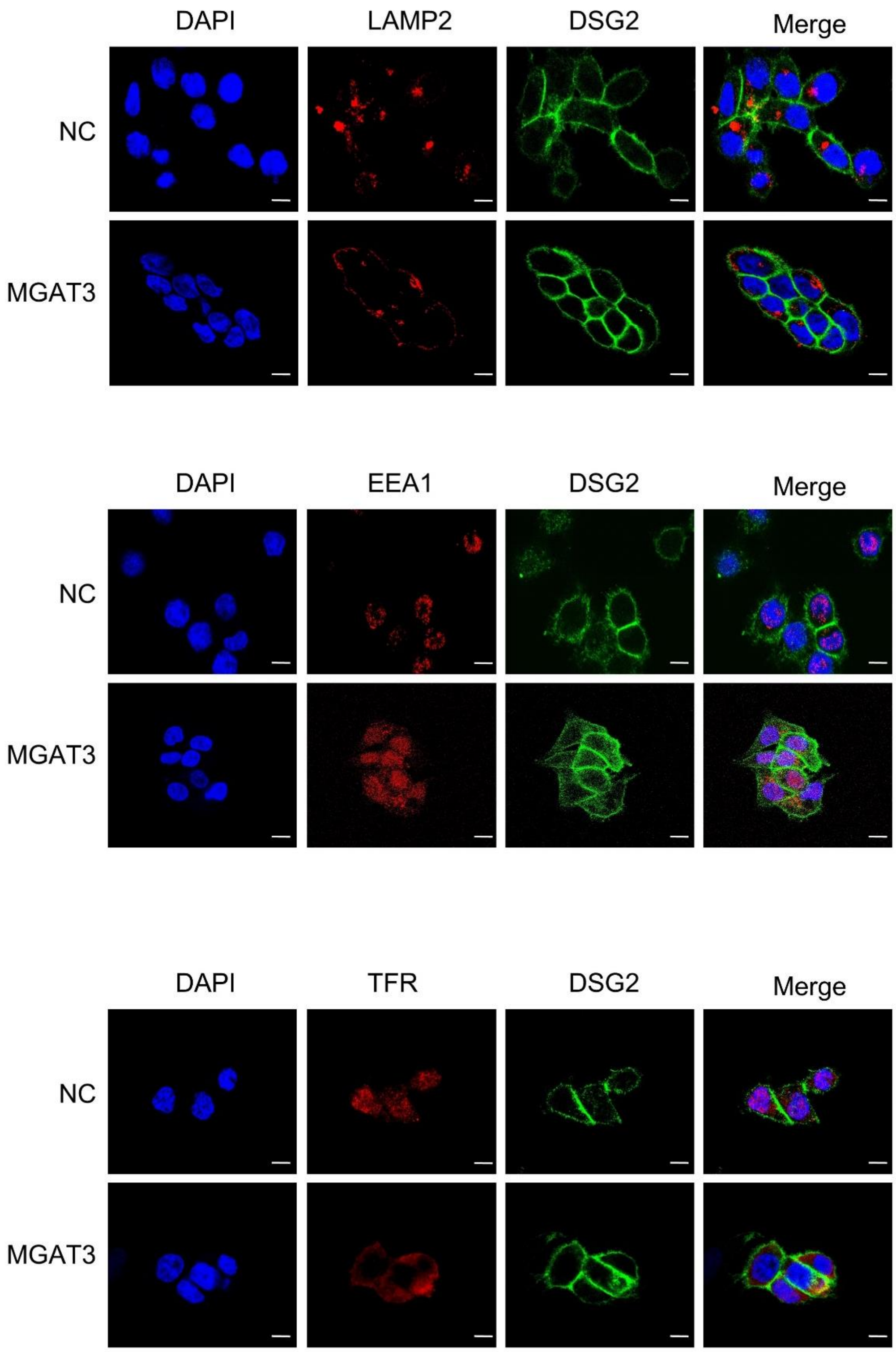

Figure S6. MGAT3 overexpression decreased DSG2 colocalization with lysosomes while increasing colocalization with early and recycling endosomes. DSG2 immunofluorescence relative to the lysosomal marker LAMP2 （up）, the early endosome marker EEA1 (middle), and the recycling endosome marker TFR staining (down, scale bars: 10 μm).

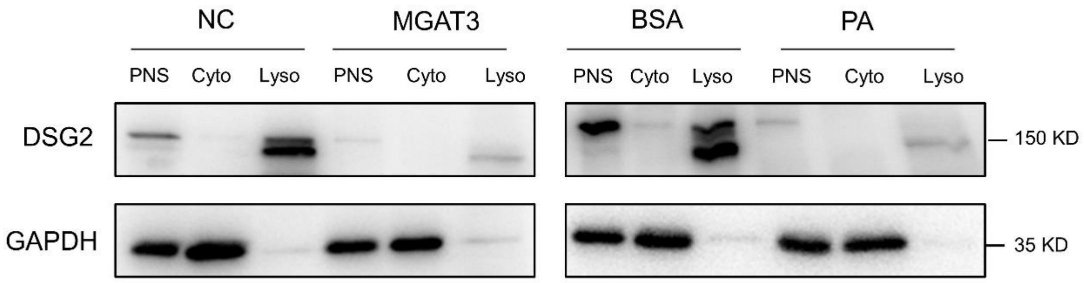

Figure S7. MGAT3 overexpression and PA treatment reduced DSG2 accumulation in lysosome-enriched fractions. Western blot of subcellular fractionations. PNS: post nuclear supernatant, Cyto: cytosol, Lyso: lysosome.

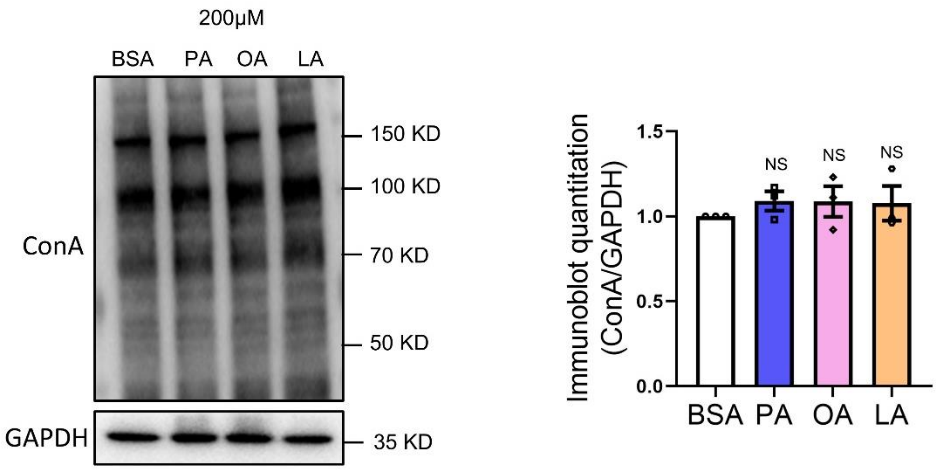

Figure S8. Global N-glycosylation levels were not altered in CRC cells treated with PA, OA and LA. Global N-glycosylation assessed by lectin blot with ConA (left). Quantification of three independent experiments (right).

SUPPLEMENTARY METHODS

Cell lines and stable transfection

Human normal colon epithelial cell lines (NCM460 and HCoEpiC) and CRC cell lines (HT29, SW480, HCT116, Sw620 and LoVo) were obtained from the Cell Bank of Chinese Academy of Sciences (Shanghai, China). NCM460, HCoEpiC, HCT116 and SW620 cells were cultured in DMEM (Gibco, CA, USA), while HT29, SW480, and LoVo cells were cultured in RPMI 1640 Medium (Gibco, CA, USA) at 37°C in a 5% CO_2 atmosphere.

MGAT3 cDNA was inserted into the pLVX lentiviral vector. Short hairpin RNAs (shRNAs) targeting MGAT3 were inserted into the PLKO.1 lentiviral vector. Vectors co-transfected with pMD2.G and psPAX2 into HEK293T cells for lentivirus production. Subsequently, SW480 and HCT116 cells were infected with lentivirus. Stable transfectants were selected and confirmed by western blotting.

H&E staining and immunohistochemistry

Mouse colon tissues were fixed in formalin and embedded in paraffin, followed by slicing into 5-μm sections. TMAs or mouse colon sections were dewaxed in xylene and rehydration. For H&E staining, sections were stained with hematoxylin and eosin (Sigma Aldrich, MO, USA). For immunohistochemistry (IHC), sections were blocked with 5% BSA in PBS for 1 h at room temperature (RT), incubated with primary antibodies against MGAT3 (cat #A8134, ABclonal, Wuhan, China,1:100) and proliferating cell nuclear antigen (PCNA, cat #13110, Cell Signaling Technology, MA, USA,1:100) or biotin-conjugated PHA-E (cat #B-1125, Vector Labs,1:50) overnight at 4°C, incubated with secondary antibody for 1 h at 37°C, visualized with DAB Detection Kit (Abcam, MA, USA), stained with hematoxylin for 1 min, and imaged using a light microscope (Nikon Instruments, Tokyo, Japan).

Protein extraction and Western blotting analysis

HCT116 or SW480 cells were treated with 200 μM PA, OA, LA or BSA for 48 h. Then the cells were washed and lysed in RIPA buffer containing protease inhibitors. Protein concentrations were determined using the BCA assay kit (Beyotime, Jiangsu, China). Proteins (20 μg) were separated by SDS-PAGE and transferred onto polyvinylidene difluoride (PVDF) membranes (Millipore, Darmstadt, Germany). The membranes were blocked with 3% BSA in TBST for 1 h at RT, incubated overnight at 4°C with primary antibodies targeting MGAT3(cat #A8134, ABclonal, Wuhan, China, 1:500), Dsg2 (cat #sc-365856, SANTA CRUZ, CA, USA, 1:500), EGFR (cat #sc-373746, SANTA CRUZ, CA, USA, 1:500), phospho-EGFR (cat #sc-377547, SANTA CRUZ, CA, USA, 1:500), AKT (cat #4685, Cell Signaling Technology, MA, USA, 1:1000), phospho-AKT (cat #4060, Cell Signaling Technology, MA, USA, 1:500), ERK (cat #4695, Cell Signaling Technology, MA, USA, 1:1000), phospho-ERK (cat #4696, Cell Signaling Technology, MA, USA, 1:500), STAT3 (cat #9139, Cell Signaling Technology, MA, USA, 1:1000), phospho-STAT3 (cat #9145, Cell Signaling Technology, MA, USA, 1:500), PI3K (cat #4292, Cell Signaling Technology, MA, USA, 1:1000), phospho-PI3K (cat #4228, Cell Signaling Technology, MA, USA, 1:500), GAPDH (cat #AC033, ABclonal, Wuhan, China, 1:5000), Actin (cat #AF8279, R&D SYSTEMS, Minneapolis, USA, 1:5000), followed by appropriate secondary antibodies.

For lectin blotting, the membranes were probed with biotin-conjugated PHA-E (cat #B-1125, Vector Labs, 1:100) overnight at 4 °C, then incubated with a VECTASTAIN Elite ABC kit (cat #KT6100, Vector Labs) for 1 h at RT. Protein bands were visualized by enhanced chemiluminescence (Vazyme Biotech, Nanjing, China), and imaged using a bioluminescence system (Tanon, Shanghai, China).

Immunoprecipitation (IP)

Cell lysates (1 mg) were incubated with 2 μg anti-Dsg2 antibody for 1 h at 4 °C. The complexes were captured using 20 μl protein A/G Plus-Agarose (Beyotime, Haimen, China) with rotation overnight at 4 °C. Beads were washed with PBS, resuspended in SDS loading buffer, and analyzed by western blotting.

Immunofluorescence staining

Cells were cultured on confocal dishes, washed with cold PBS, fixed with 4% paraformaldehyde for 15 min, permeabilized with 0.2% Triton X-100 for 10 min, blocked with 3% BSA for 2 h at RT, incubated with anti-Dsg2 antibody (cat #sc-365856, SANTA CRUZ, CA, USA, 1:200) overnight at 4 °C, then incubated with FITC-conjugated anti-mouse IgG antibody (cat #ab6717, Abcam, Cambridge, UK, 1:500) for 2 h at RT, washed 3 times with PBS, and incubated with DAPI for 15 min at RT. Images were captured by fluorescence microscopy (Leica, Mannheim, Germany). For lectin staining, fixed and blocked cells were incubated with FITC-conjugated PHA-E lectin (Vector Labs, 1:100) and analyzed by fluorescence microscopy or flow cytometry (ACEA Biosciences, CA, USA). Three random fields with cell homogeneity on the glass slide were analyzed and quantified using ImageJ software. The fluorescence intensity was calculated as the total fluorescence intensity divided by the number of nuclei. The background intensity was subtracted using ImageJ to ensure accuracy. The experiments were performed in triplicate, and statistical analysis was conducted using one-way ANOVA.

Quantitative real-time PCR (qRT-PCR)

Total RNA was isolated from tissues or cells based on manufacturer's instructions of TRIzol reagent. The

concentration and quality of the extracted RNA were measured by NanoDrop Spectrophotometer (Thermo, CA, USA). The RNA was then reverse-transcribed into cDNA using HiScript II Q RT SuperMix (Vazyme Biotech, Nanjing, China). The qRT-PCR analyses were performed using the same amount of DNA (5 ng/mL) with AceQ qPCR SYBR Green Master Mix (Vazyme Biotech, Nanjing, China). Primer sequences are listed in **Supplementary Table S5**. Gene expression were quantified by $2^{-\Delta\Delta}$Ct method, normalized to GAPDH.

Cell apoptosis

After 48 hours of treatment, cells were harvested, and then washed twice with PBS. Cells were resuspended in 100μL of binding buffer with 2.5 μL of Annexin V-FITC and 2.5 μL of 7-AAD (APC Annexin V Apoptosis Detection Kit with 7-AAD, BioLegend, CA, USA). After incubation for 15min in the dark at 25 °C, an additional 200 μL of binding buffer was added to each tube. Finally, the cells were analyzed by flow cytometry (ACEA Biosciences, CA, USA).

Proliferation

For Edu assay, HCT116 cells were incubated with 10 μM Edu for 3 h, fixed with 4% paraformaldehyde, permeabilized with 0.2% Triton X-100, stained with iClick EdU solution (ABP Biosciences), rinsed with PBS, and analyzed by flow cytometry (ACEA Biosciences, CA, USA). For the Cell Counting Kit-8 (CCK-8) assay, HCT116 cells in 96-well plates were incubated with CCK8 solution (TopScience; Shanghai, China) for 3 h at 37 °C. Absorbance at 450 nm was measured using a microplate reader (Bio-Rad Laboratories, CA, USA).

Transwell assay

Cell migration or invasion was assessed using the Transwell assay. For the migration, $2×10^4$ CRC cells were starved in serum-free medium for 12 h, and then seeded in the upper chambers (pore size 8 *μ*m; Corning, NY, USA). Complete medium was added to the bottom chamber. After 48 h at 37 °C, migrated cells were fixed with 4% paraformaldehyde for 15 min, stained with 0.1% crystal violet for 10 min, and photographed under a light microscope. For the invasion assay, the upper chambers were coated with 50 μl of Matrigel (Corning, NY, USA) before seeding cells.

Lysosomal protein extraction

HCT116 cells were serum starved for 2 hours before harvest. The cells were resuspended in Reagent A (Solarbio #EX1230), incubated on ice for 10 minutes with shaking, and then homogenized using a Dounce homogenizer until approximately 90% cell lysis was achieved. The homogenate was centrifuged at 1,000 g for 5 min at 4°C to remove large cell debris and nuclei, and the supernatant is post-nuclear supernatant (PNS). PNS was transferred to a new centrifuge tube and centrifuged at 20,000 g for 20 min at 4°C to pellet down the crude lysosomal fraction, and the supernatant is cytosolic fraction. The pellet was washed with 500 μL of ice-cold Reagent B and centrifuged again at 20,000 g for 20 min at 4°C. The final pellet was lysed in Extraction Buffer C (supplemented with protease inhibitors) by gentle agitation for 30 min at 4°C. Following centrifugation at 12,000 g for 15 min at 4°C, the resulting supernatant contained the lysosomal protein fraction.

Supplementary Table 1. Differentially expressed proteins identified in colon tissues of AOM/DSS-induced CRC mice compared with control mice by proteomics analysis

Gene symbol	-log$_{10}$*p*value	*p*value	log$_2$fold change	fold change
St3gal4	1.87894	0.013215	-3.12783	0.114401
Snf8	1.47877	0.033207	-3.07633	0.118558
Fmo2	1.71712	0.019181	-2.94049	0.130264
Scyl1	2.04955	0.008922	-2.63352	0.16115
Otc	1.36005	0.043647	-2.62019	0.162646
Uqcrb	1.96267	0.010898	-2.33225	0.198574
Entpd2	1.54955	0.028213	-2.27982	0.205923
Pgrmc1	1.69328	0.020264	-2.2453	0.21091
Chl1	1.42173	0.037868	-2.14953	0.225386
Ppp6c	1.30302	0.049771	-2.12451	0.229329
Agl	1.44933	0.035536	-2.04609	0.242139
Gaa	1.8082	0.015552	-1.96582	0.255994
Eif1	2.0248	0.009445	-1.93287	0.261908
Mtx2	1.31835	0.048045	-1.92384	0.263552
Pygm	1.94408	0.011374	-1.92192	0.263903
Sgsh	1.60019	0.025108	-1.83575	0.280146
Aarsd1	1.9118	0.012252	-1.78838	0.289497
Scin	2.10898	0.007781	-1.78618	0.289939
Abat	1.48976	0.032377	-1.75342	0.296598

Pnpt1	1.50658	0.031147	-1.74043	0.29928
Tppp	1.39395	0.040369	-1.73156	0.301126
Nutf2	2.12629	0.007477	-1.72905	0.301651
Prpsap2	2.07629	0.008389	-1.71722	0.304134
Hdhd2	1.30895	0.049096	-1.69447	0.308968
Sprr2a	1.60197	0.025005	-1.67336	0.313522
Atp6v1h	1.34174	0.045526	-1.65384	0.317793
Itga3	1.82595	0.01493	-1.59175	0.331769
Tm9sf4	2.07879	0.008341	-1.55621	0.340043
Selm	1.90194	0.012533	-1.55113	0.341243
Fam129b	2.16917	0.006774	-1.54946	0.341638
Prkar2a	2.31832	0.004805	-1.47952	0.358608
Tep1	1.36882	0.042774	-1.44795	0.366542
Rab5a	1.5072	0.031103	-1.31892	0.400835
Tfrc	1.40907	0.038988	-1.30034	0.40603
Cth	1.91445	0.012177	-1.26924	0.414878
Slc4a7	1.66044	0.021855	-1.26104	0.417243
Rab5b	1.44347	0.036019	-1.26086	0.417295
Fkbp11	1.46221	0.034498	-1.24879	0.420801
Ddx39b	1.6298	0.023453	-1.19684	0.43623
Prkacb	1.78689	0.016335	-1.14831	0.451153
Eif3i	1.63655	0.023091	-1.14362	0.452622

Ugt2b34	1.60603	0.024773	-1.11694	0.461071
Akr1e2	1.39777	0.040016	-1.11301	0.462328
Dnajb4	1.46928	0.033941	-1.07988	0.473068
Dpysl3	1.78977	0.016227	-1.06207	0.478944
Erlec1	1.57717	0.026475	-1.02705	0.490713
Ccdc43	1.43972	0.036331	-0.99329	0.502332
Etf1	1.7997	0.01586	-0.97461	0.508877
Hsd17b11	2.21703	0.006067	-0.95803	0.514759
Ndufv1	2.08719	0.008181	-0.94375	0.519879
Cbs	1.45362	0.035187	-0.94348	0.519979
Coq9	1.34207	0.045491	-0.9376	0.522102
Mzt2	1.59583	0.025361	-0.89714	0.536952
Uqcrfs1	3.022	0.000951	-0.86866	0.547657
Eif4e	1.42366	0.0377	-0.85655	0.552272
Cryl1	1.39166	0.040583	-0.84815	0.555497
Map1b	1.65871	0.021943	-0.84074	0.558357
Psma2	1.56865	0.026999	-0.82322	0.565178
Slmap	1.79351	0.016088	-0.78935	0.578605
Got1	1.34328	0.045365	-0.78183	0.581628
Ndufs2	1.82187	0.015071	-0.78061	0.582122
Mgat3	1.30986	0.048994	-0.77281	0.585276
Dnm1l	1.31964	0.047903	-0.75055	0.594377

Aspn	1.47692	0.033349	-0.74351	0.597283
Gstm1	2.79986	0.001585	-0.74179	0.597996
Cant1	1.64158	0.022825	-0.7412	0.598242
Stt3a	1.43692	0.036566	-0.7393	0.599029
Cd99	1.44154	0.036179	-0.71821	0.607852
Ncl	1.60247	0.024976	0.689882	1.613152
Anp32b	1.78266	0.016495	0.748021	1.679487
Ttll12	1.85743	0.013886	0.800658	1.741895
Mllt4	1.49512	0.03198	0.807222	1.749839
Engase	1.60683	0.024727	0.828954	1.776397
Mrps9	1.47783	0.033279	0.929593	1.904739
Urod	1.79859	0.0159	0.939775	1.918229
Pdhx	1.4213	0.037905	0.955532	1.939295
Lgals3bp	1.52696	0.029719	1.02884	2.040383
Gyg1	1.77838	0.016658	1.03495	2.049043
Dpp7	1.87196	0.013429	1.06145	2.087028
Nono	1.54601	0.028444	1.08545	2.122037
Glul	2.04743	0.008965	1.09521	2.136442
Ctbp1	2.46999	0.003389	1.12279	2.177677
Snapin	1.60825	0.024646	1.14345	2.209087
Lamp2	1.45968	0.034699	1.14354	2.209224
Arrb1	1.62889	0.023502	1.19129	2.283568

Smarca5	1.35057	0.04461	1.26376	2.401207
Igfbp7	1.72877	0.018674	1.267	2.406606
Prrc2a	1.33876	0.04584	1.30972	2.478934
Il1rn	1.75824	0.017449	1.32016	2.496938
Hexa	1.51015	0.030892	1.34806	2.545696
Pak1	1.43384	0.036826	1.44935	2.73085
Abcf2	1.86739	0.013571	1.51218	2.852407
Thbs1	1.56335	0.027331	1.61102	3.054677
Thop1	1.47445	0.033539	1.6251	3.084635
Lrrc16a	1.75462	0.017595	1.63758	3.111435
Ndufb5	1.75961	0.017394	1.76392	3.396197
Sub1	1.32941	0.046837	2.32915	5.025092
Lyz1	1.88349	0.013077	3.22155	9.327885
Ngp	2.26174	0.005473	5.44157	43.45861

Supplementary Table 2.xlsx

All proteins identified in colon tissues of AOM/DSS-induced CRC mice and control mice by proteomics analysis

Supplementary Table 3. xlsx

All peptide-spectrum-matches identified as intact glycopeptides from PA treated HCT116 cells and control cells by LC-MS/MS

Supplementary Table 4. Composition of the diets used in animal experiment

Ingredient (g/kg)	Control diet	PA-medium diet	PA-rich diet
Casein	200	200	200
Dyetrose	132	132	132
Cornstarch	398	398	398
Sucrose	100	100	100
Cellulose	50	50	50
Salt Mix	35	35	35
Vitamin Mix	10	10	10
L-Cystine	3	3	3
Choline Bitartrate	2.5	2.5	2.5
Corn Oil	70	35	7
Palm Oil	0	35	63
**Fatty acid (%)**			
C14:0	0	0.5	0.9
C16:0	11	28	41.6
C18:0	2	3	3.8
C18:1	28	34	38.8
C18:2 n6	58	34	14.8
C18:3 n:3	1	0.5	0.1

Supplementary Table 5. The primer sequences of detected genes

Gene	Forward	Reverse
Gapdh	AGGTCGGTGTGAACGGATTTG	TGTAGACCATGTAGTTGAGGTCA
Mgat3	ATGAAGATGAGACGCTACAAGC	CTGGATACGAGGTTAGGGCTG
Pcna	TTGCACGTATATGCCGAGACC	GGTGAACAGGCTCATTCATCTCT
Mki67	ATCATTGACCGCTCCTTTAGGT	GCTCGCCTTGATGGTTCCT
Dsg2	TTGACCGGGAAGAAACACCAT	ACTTTGATGCGTAGTTCCAAGG
GAPDH	GGAGCGAGATCCCTCCAAAAT	GGCTGTTGTCATACTTCTCATGG
MGAT3	ATGAAGATGAGACGCTACAAGC	GCTGGACACCAGGTTAGGG
