# Supplementary material for: Dietary palmitic acid inhibits colorectal cancer progression through enhancing bisecting GlcNAc
Source: JCI Insight. 2026 Mar 23;11(6):e179533. doi: 10.1172/jci.insight.179533 (PMC13043105; doi:10.1172/jci.insight.179533)

# Full unedited gel for Figure 1D

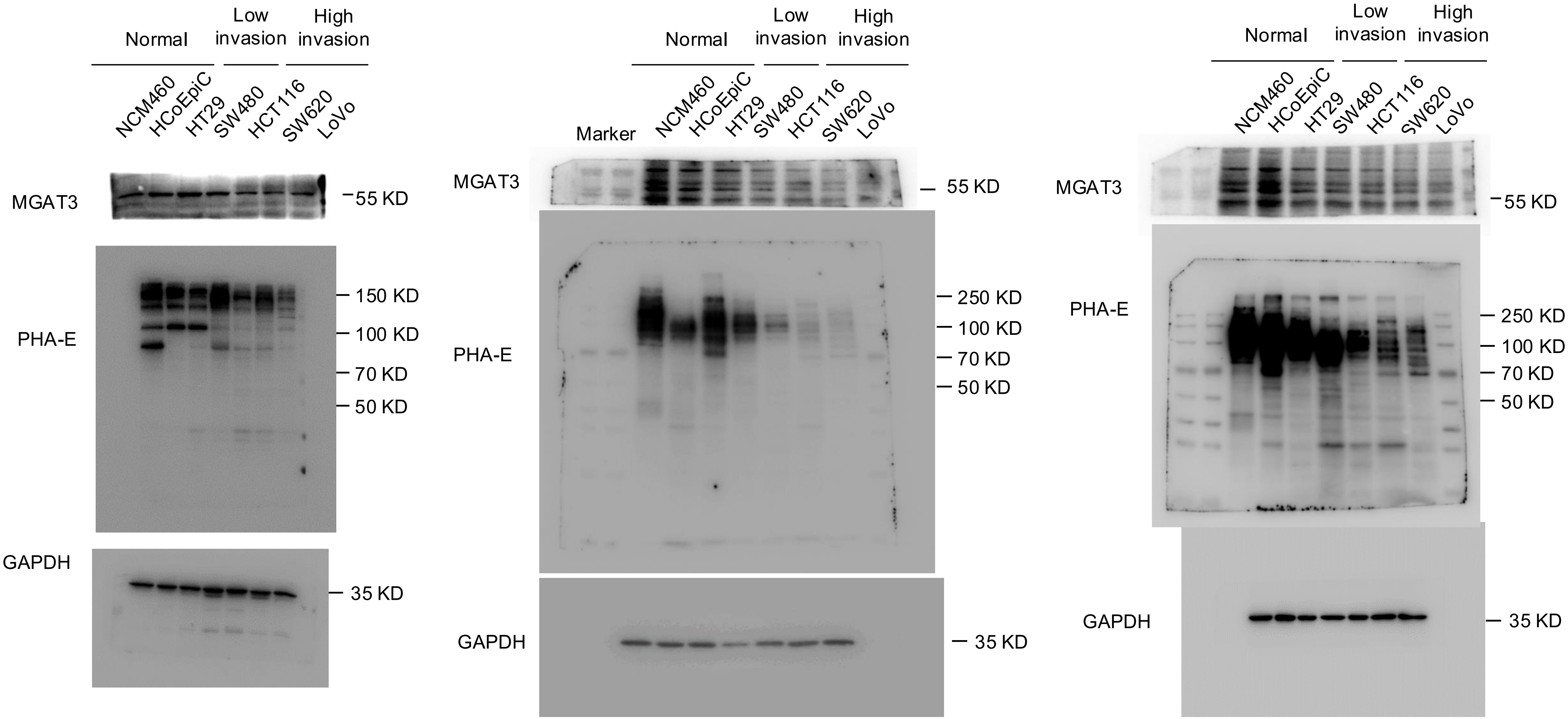

# Full unedited gel for Figure 3A

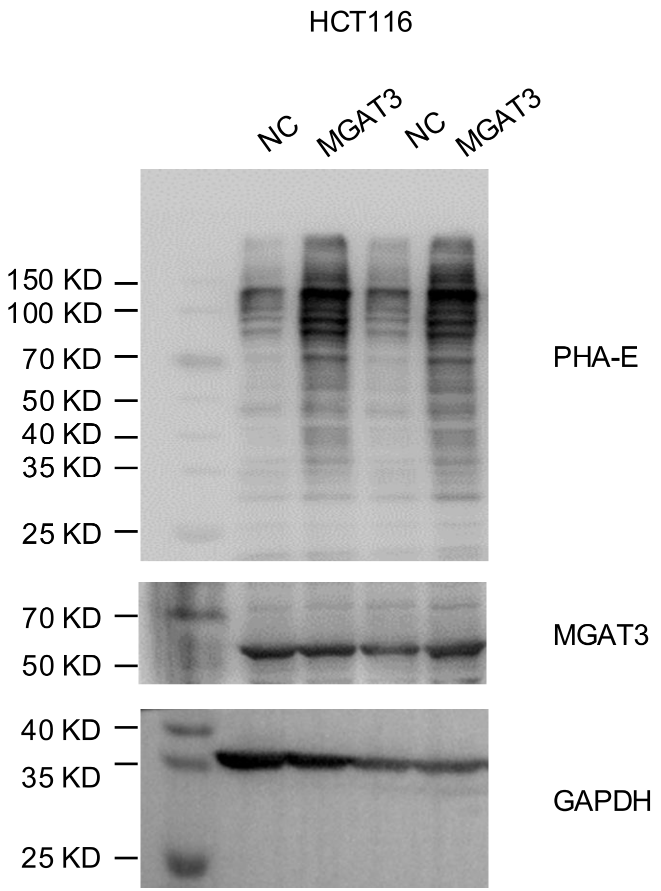


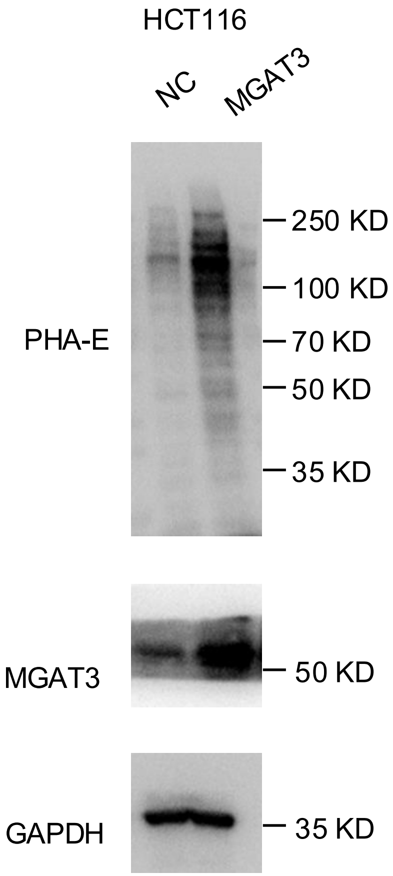

# Full unedited gel for Figure 3H

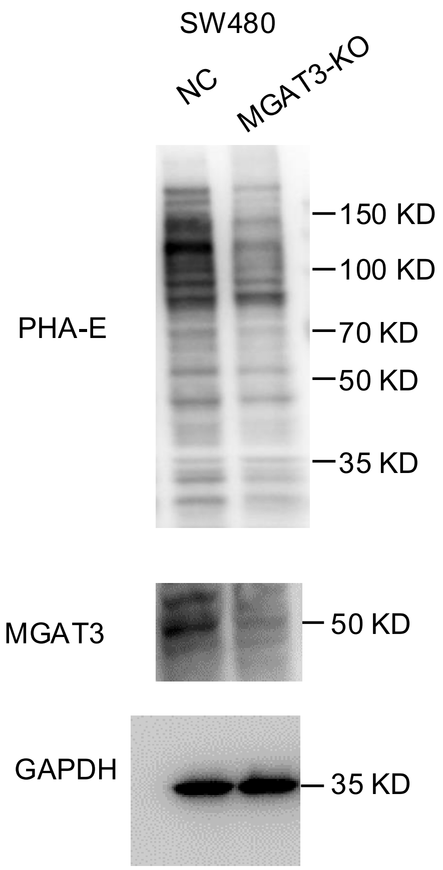


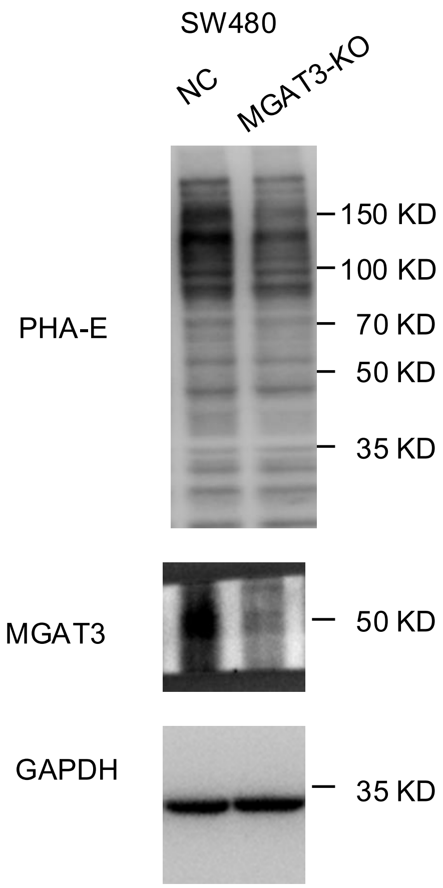


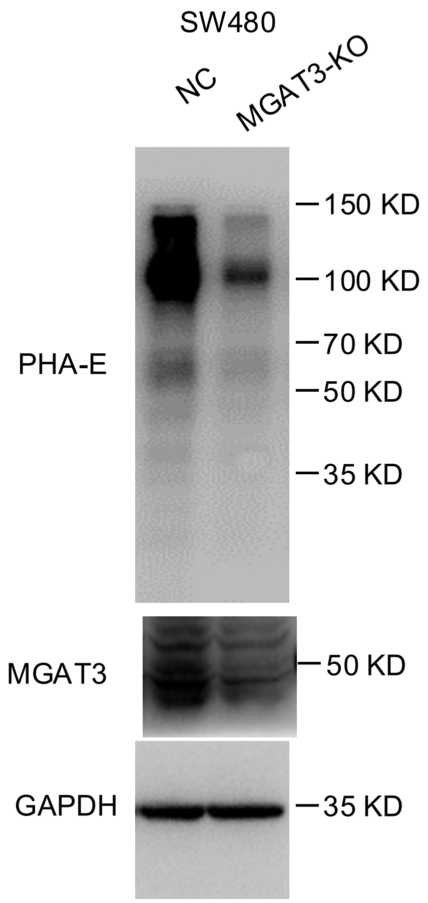

# Full unedited gel for Figure 4A

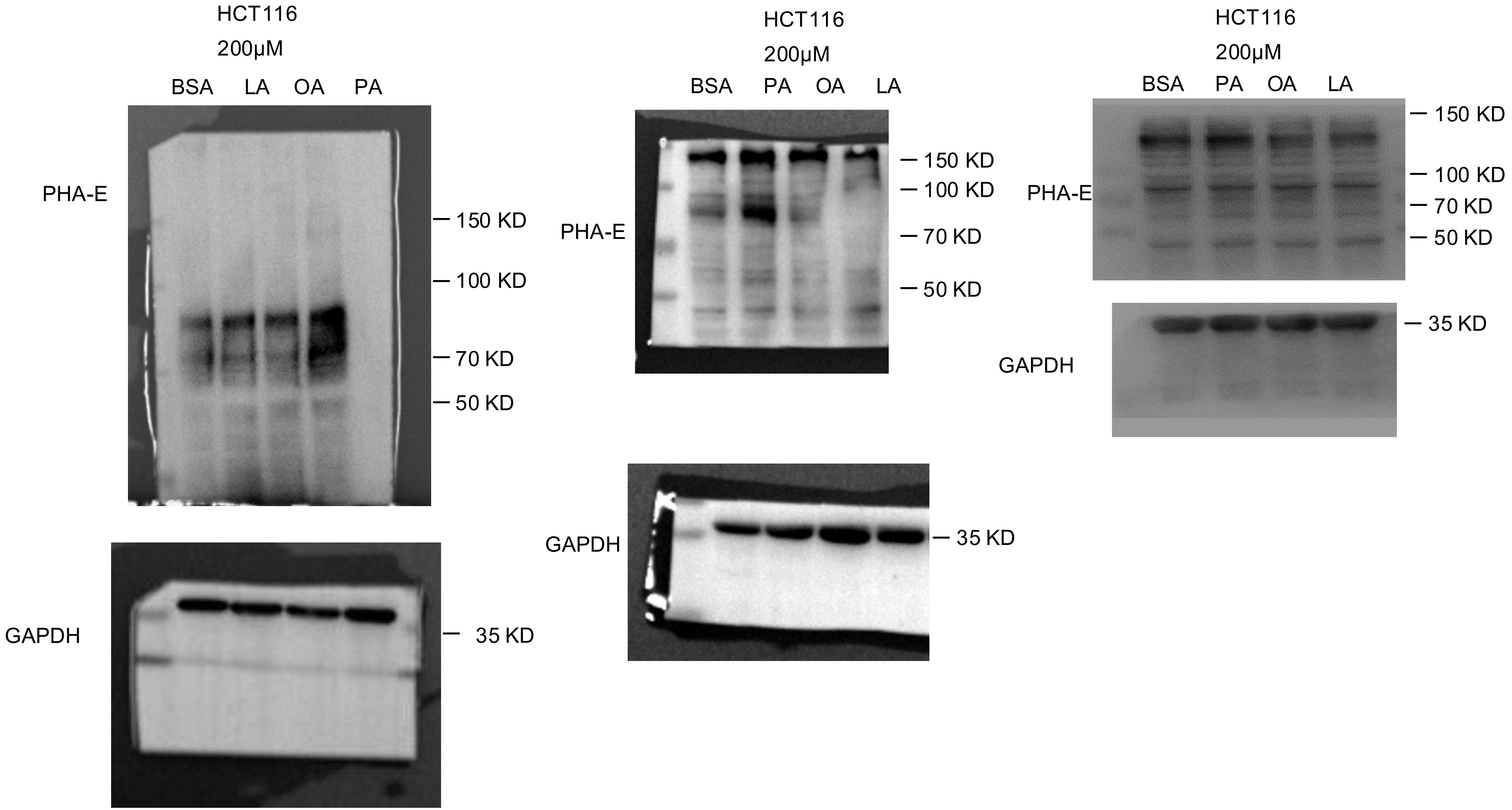

# Full unedited gel for Figure 4I

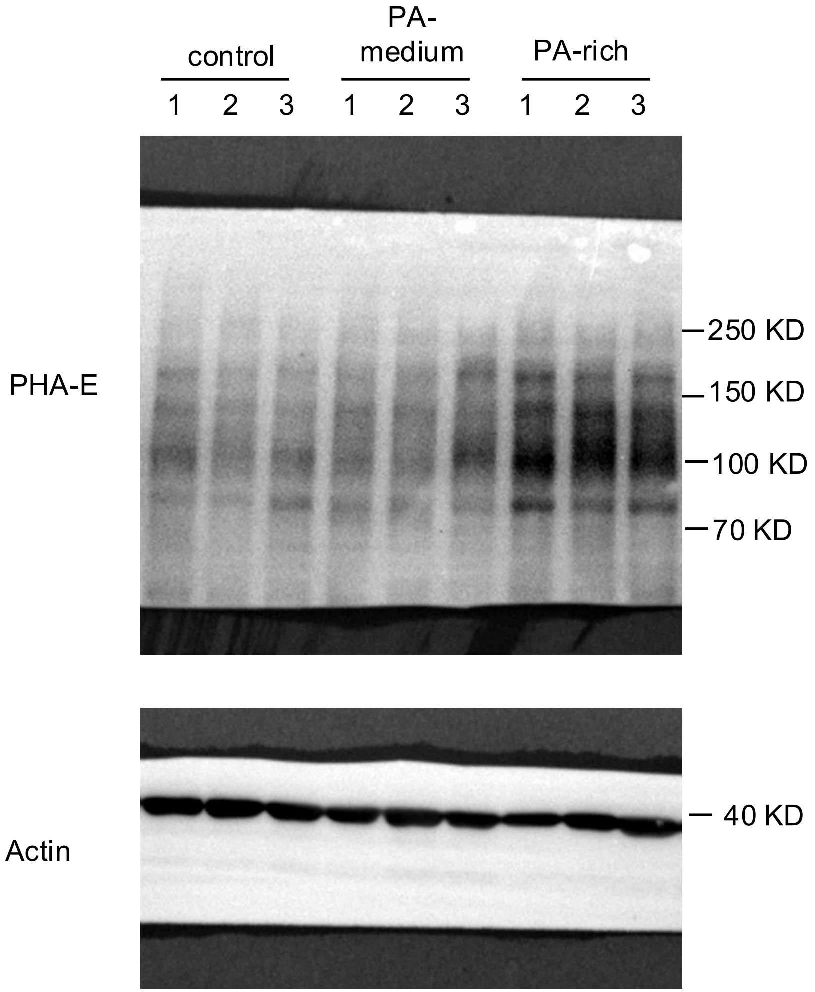

# Full unedited gel for Figure 5A

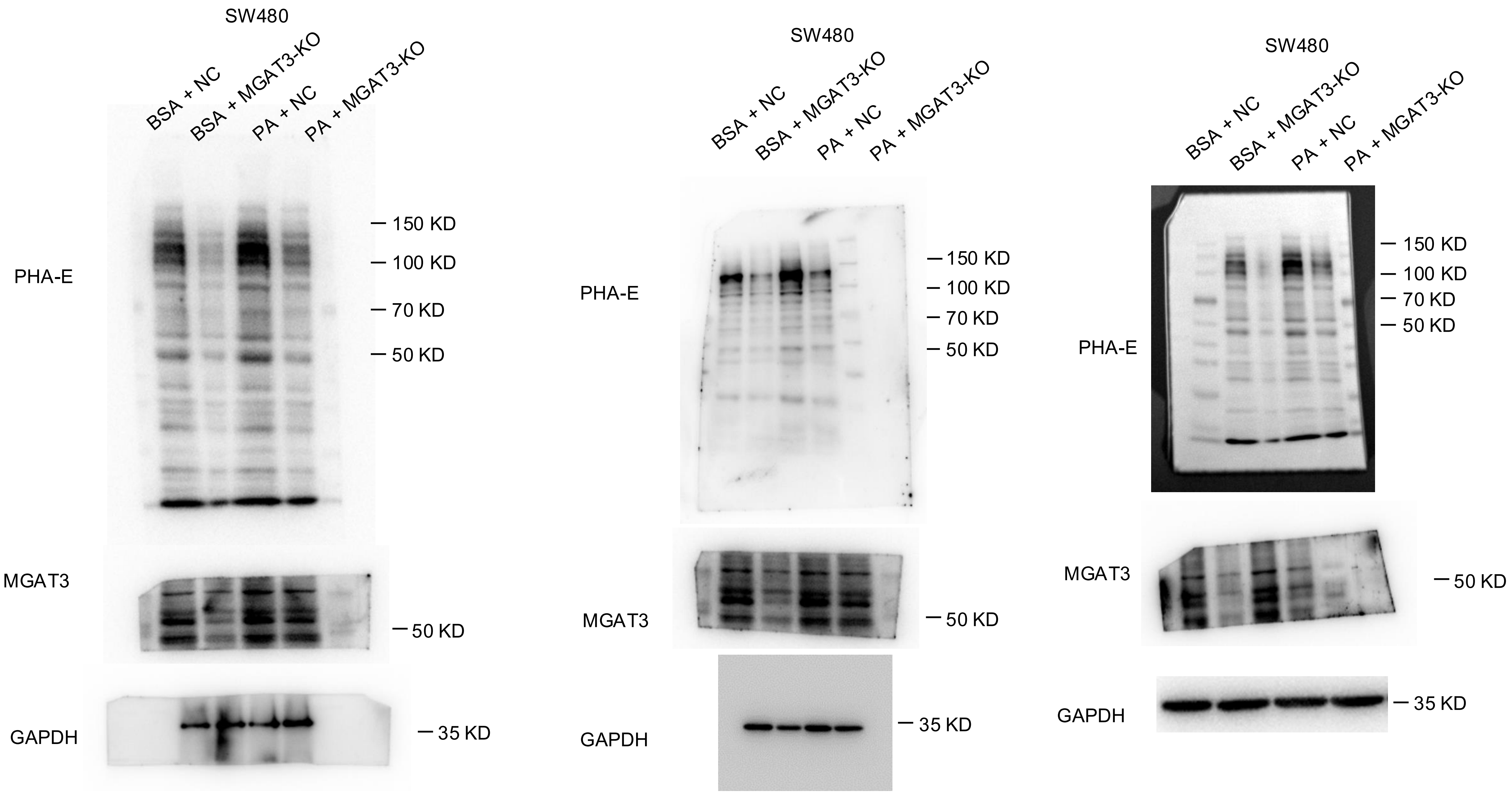

# Full unedited gel for Figure 5I

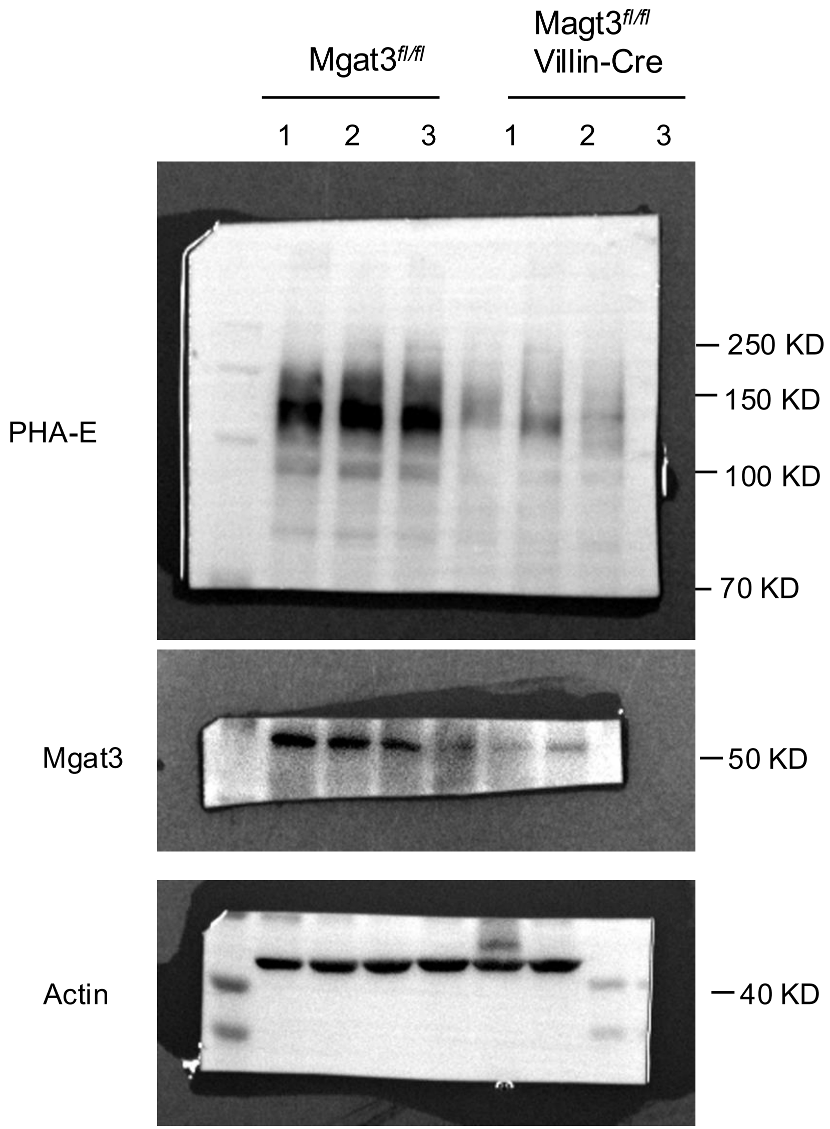

# Full unedited gel for Figure 6C

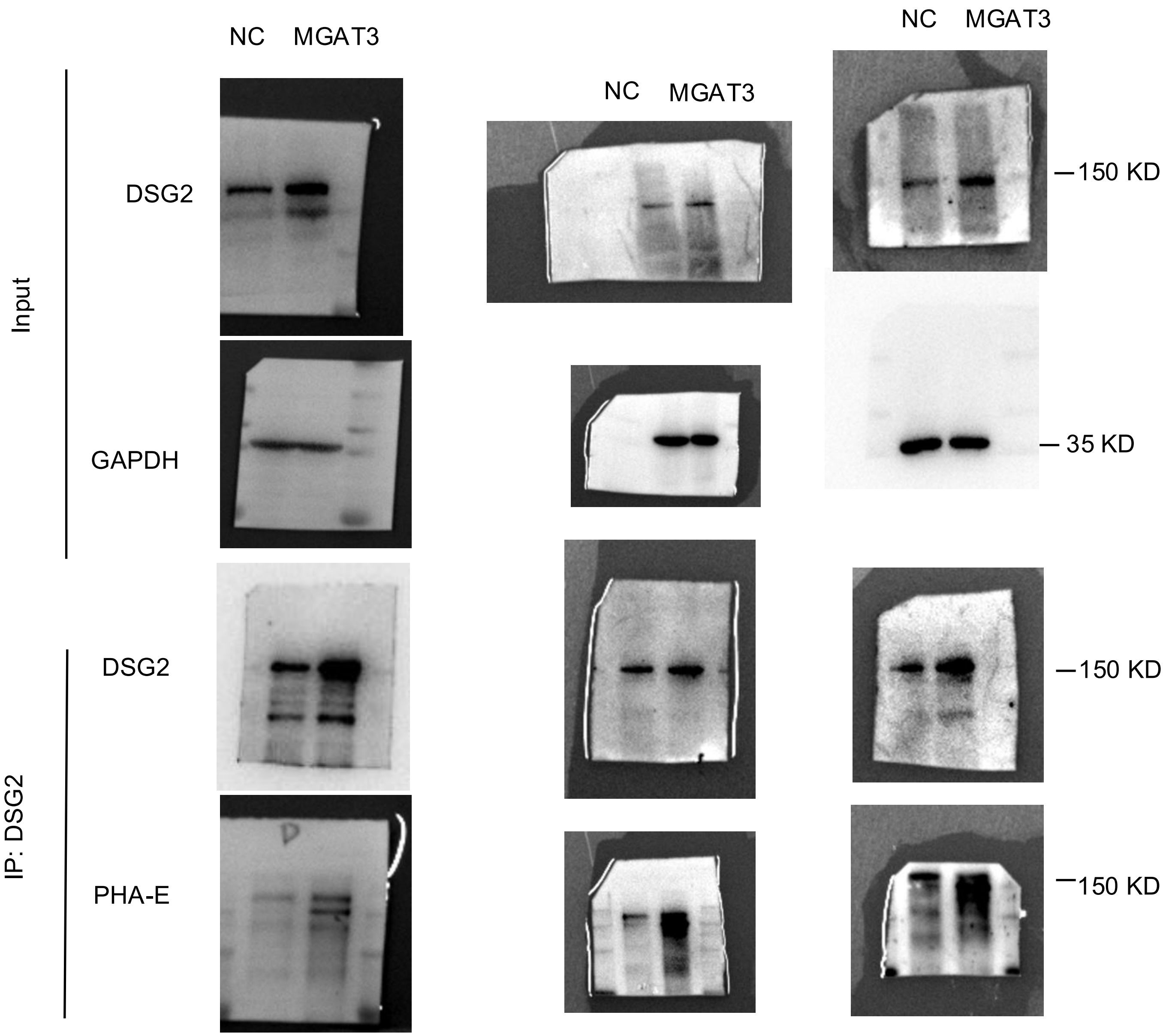

# Full unedited gel for Figure 6C

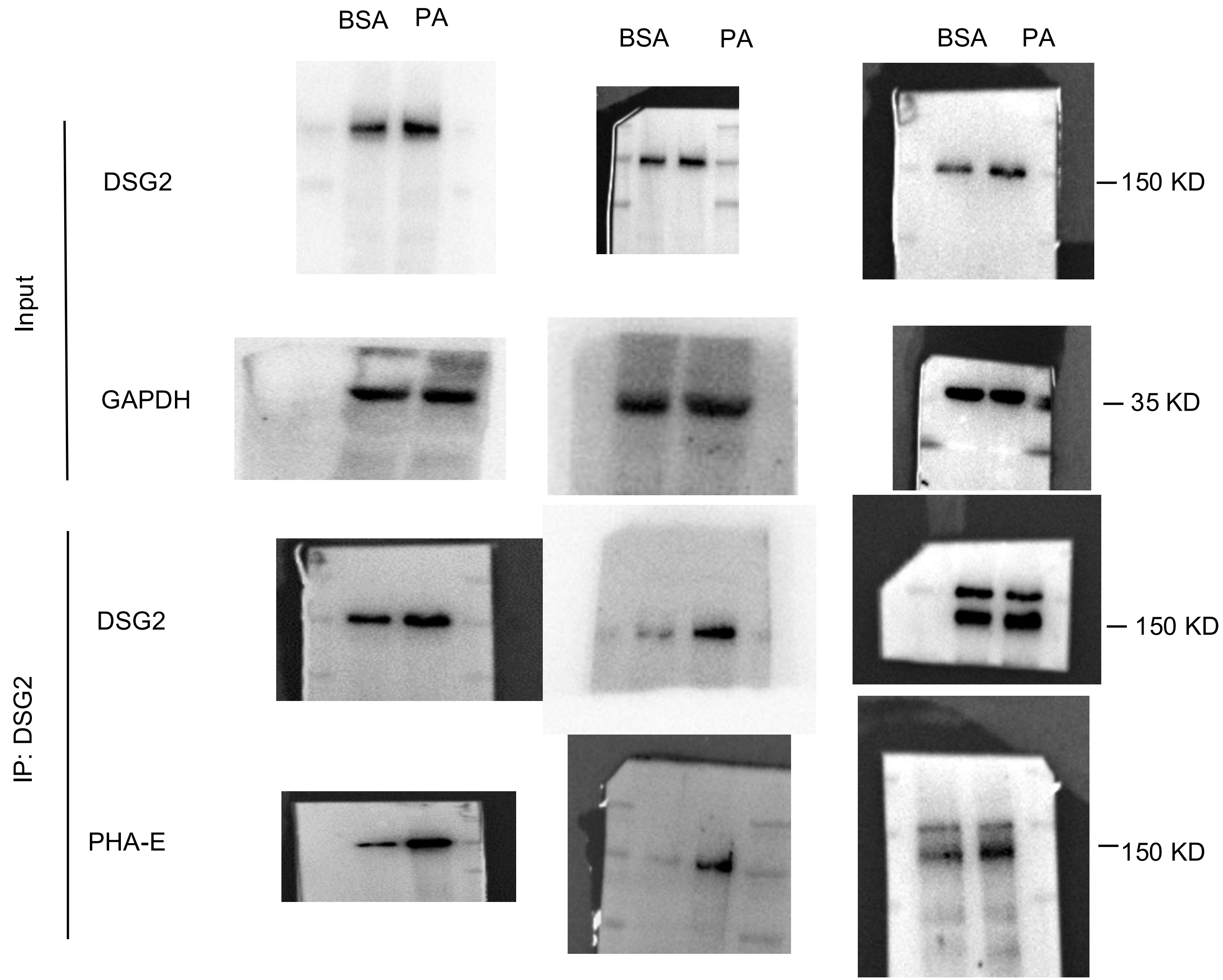

# Full unedited gel for Figure 6E

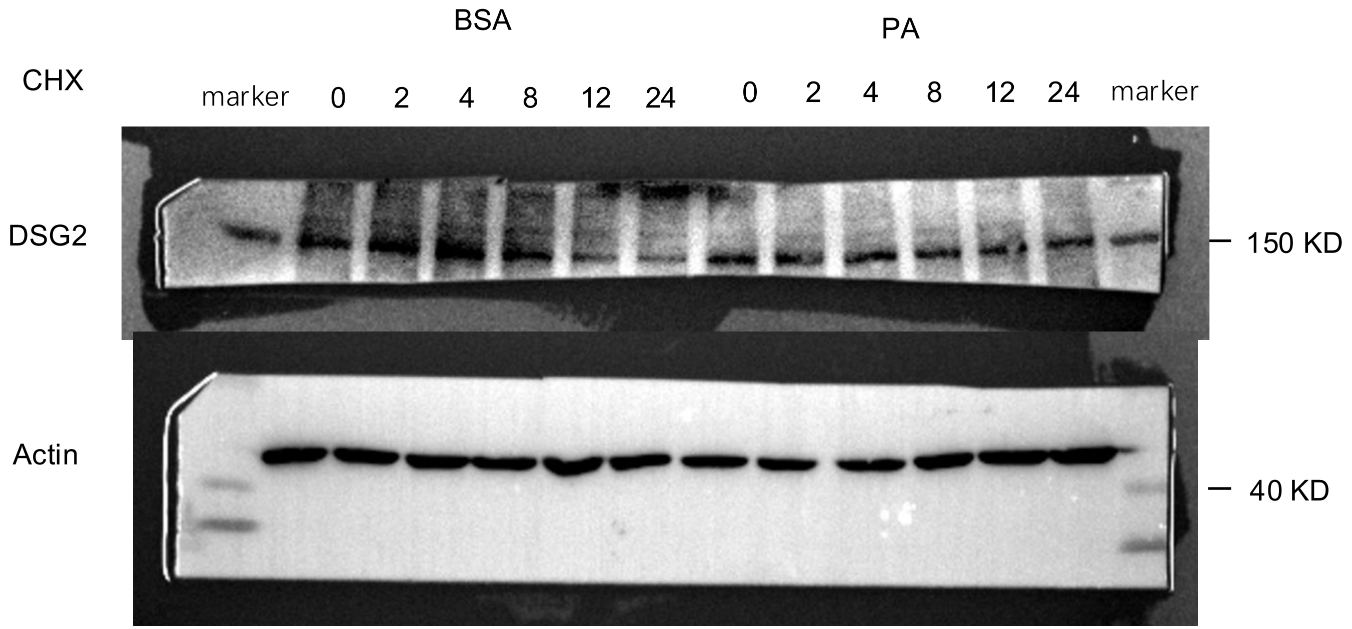


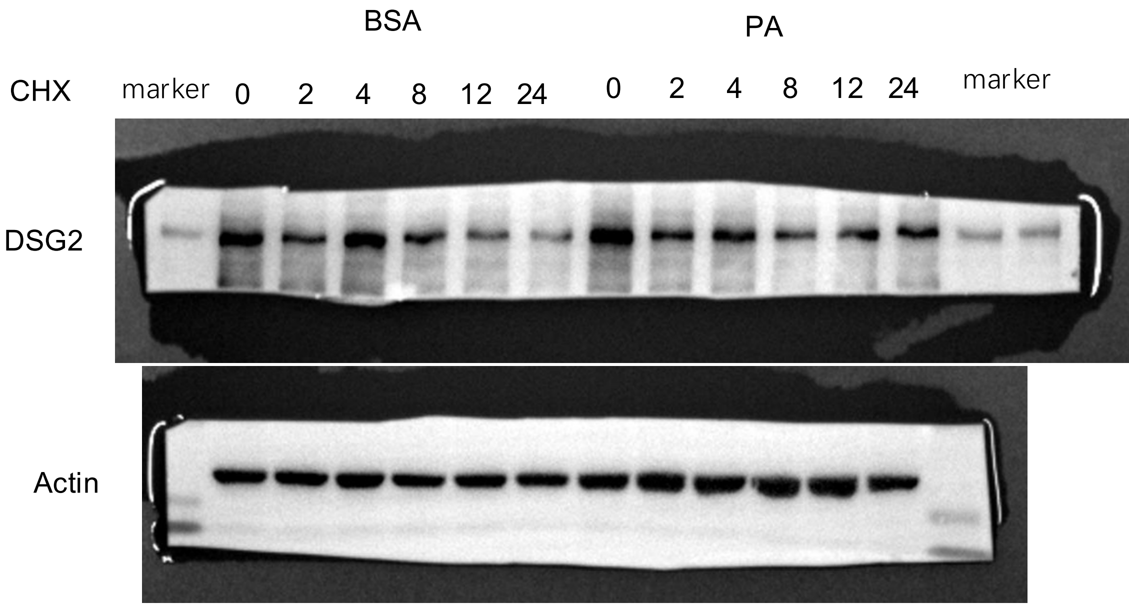


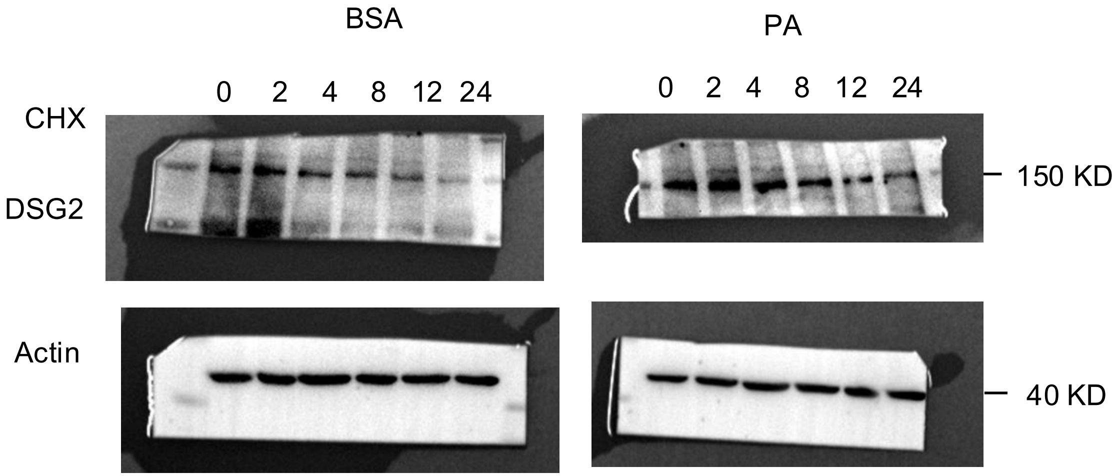

# Full unedited gel for Figure 6F

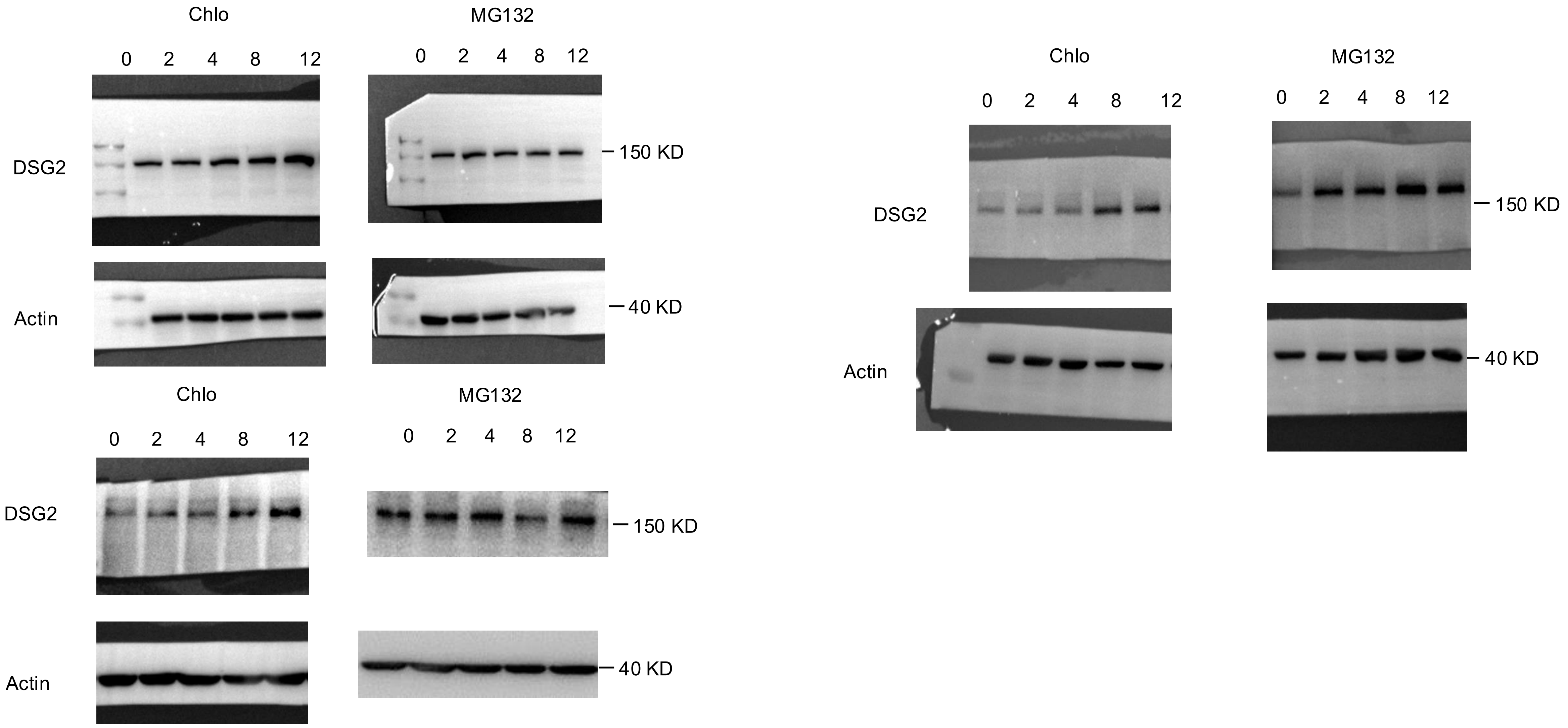

# Full unedited gel for Figure 6G

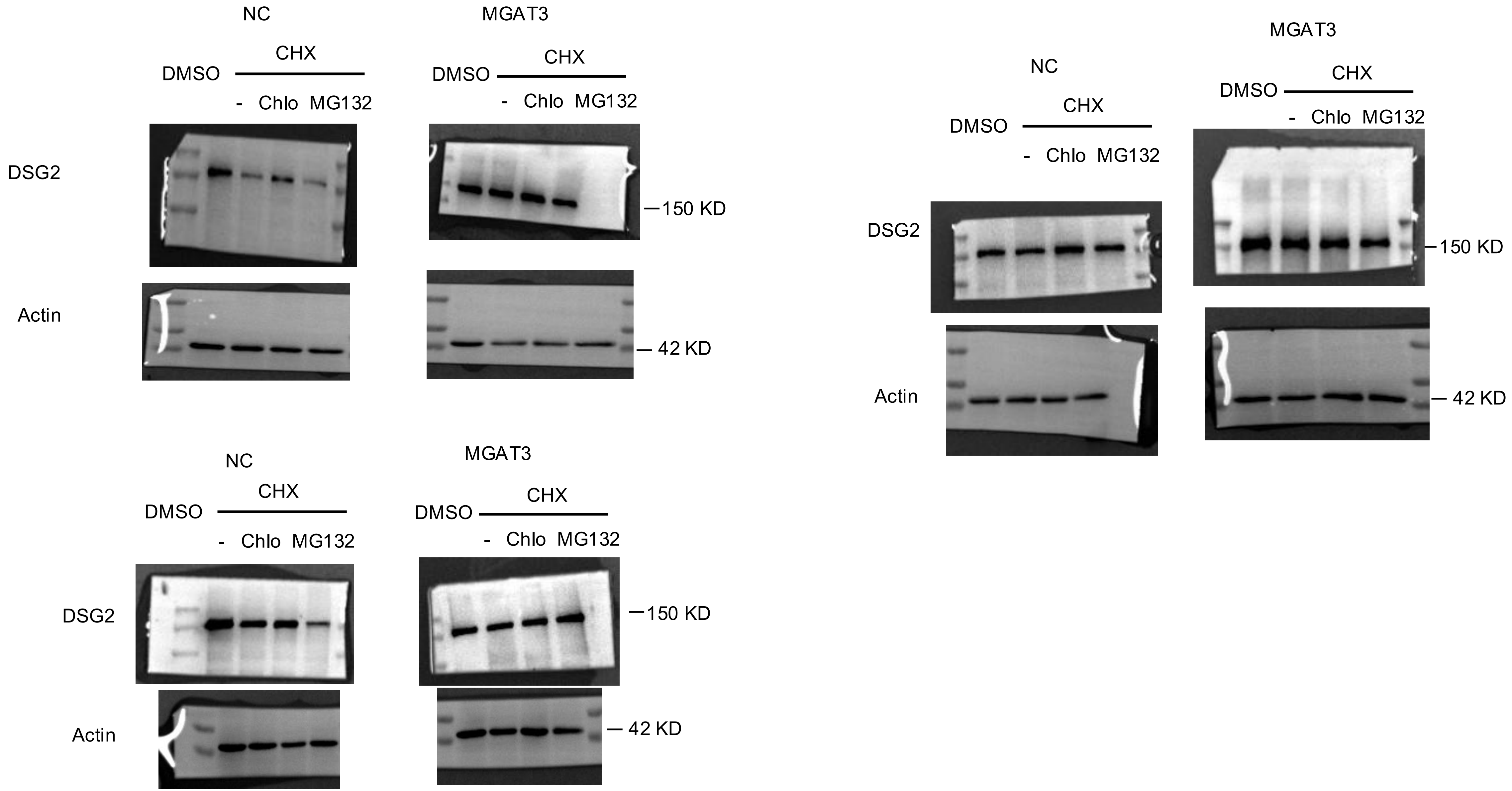

# Full unedited gel for Figure 6H

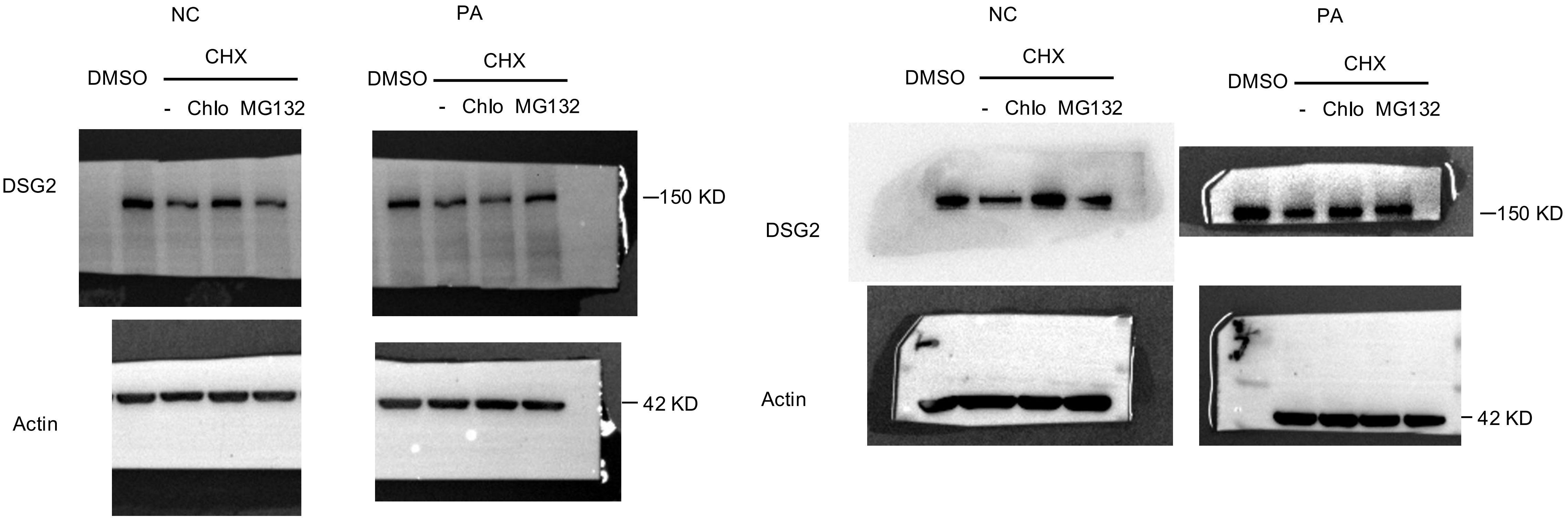

# Full unedited gel for Figure 6H

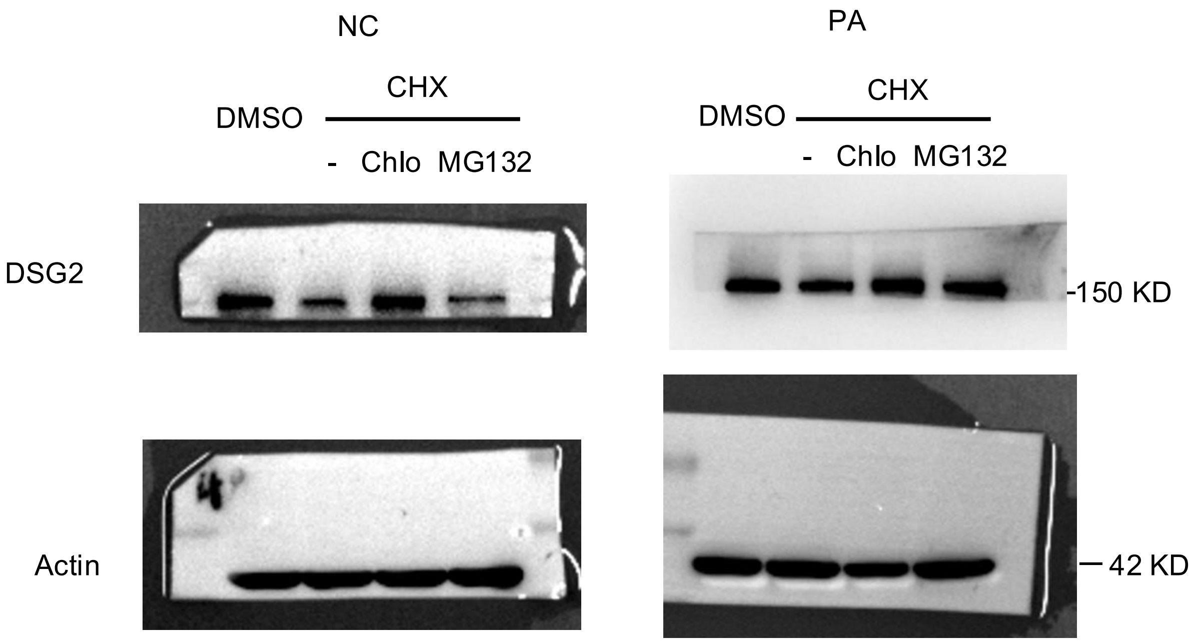

# Full unedited gel for Figure 7E

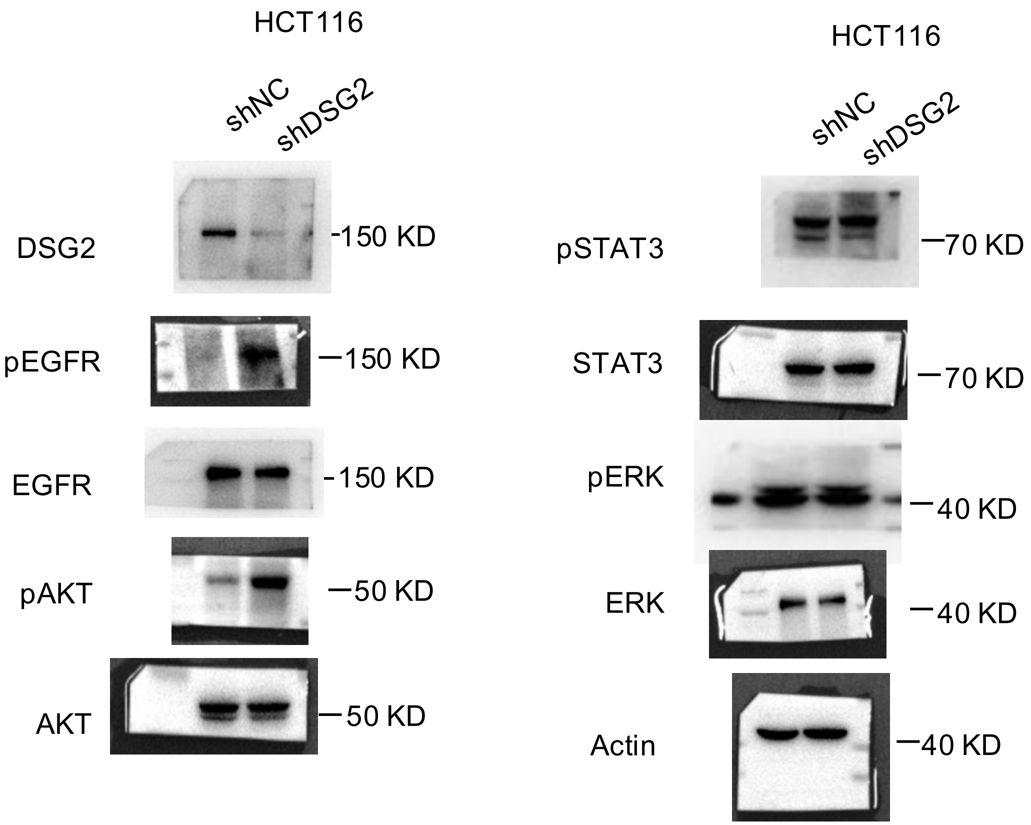

# Full unedited gel for Figure 7E

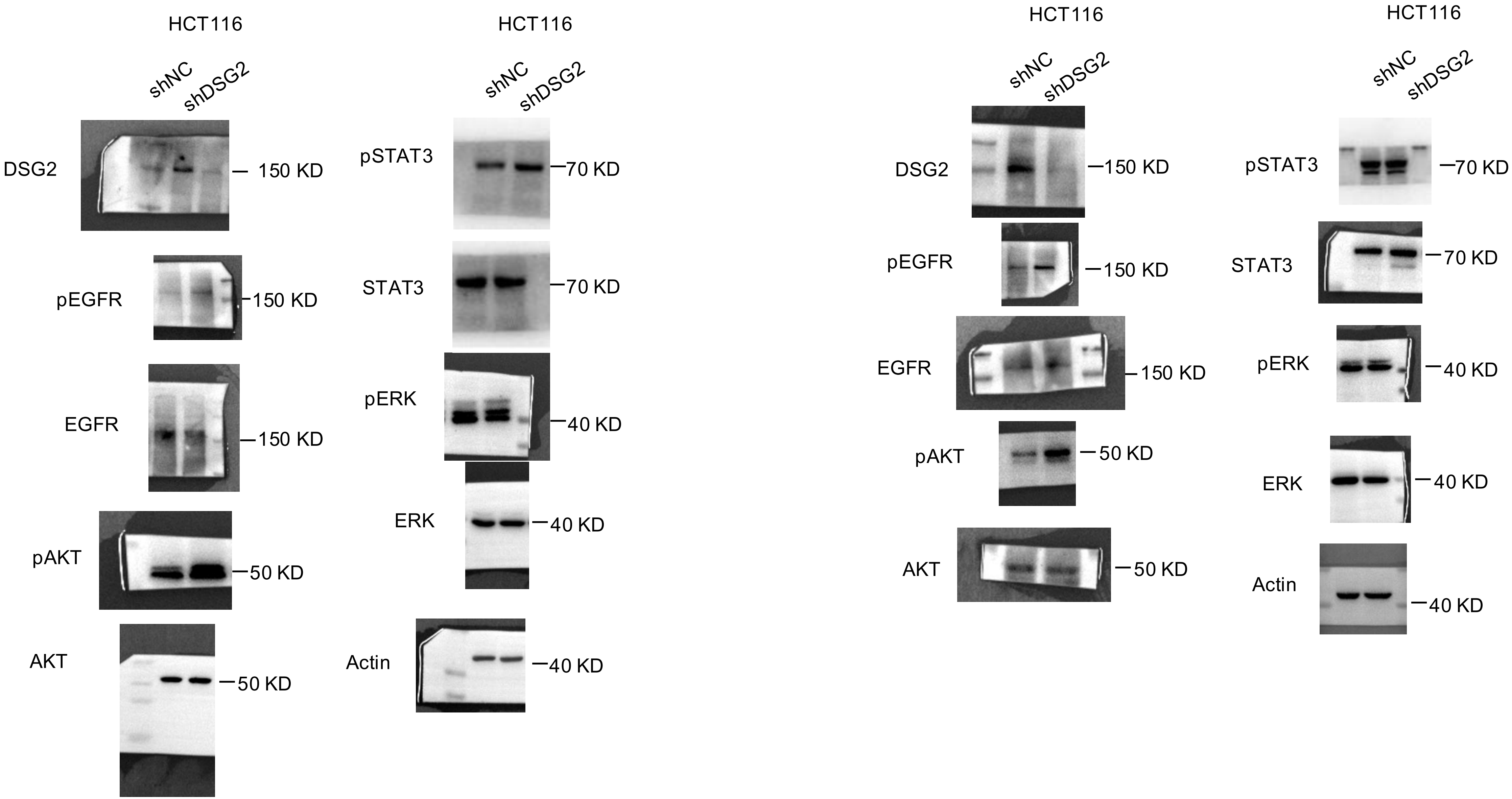

# Full unedited gel for Figure 7E

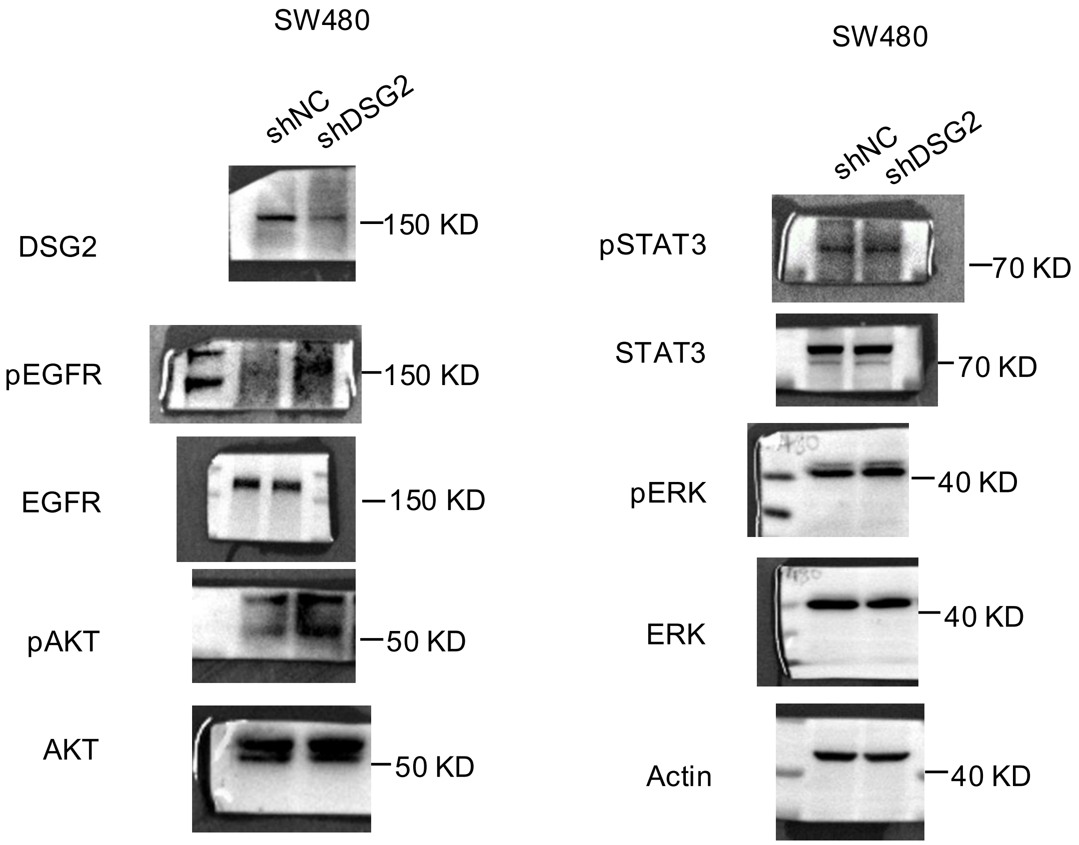

# Full unedited gel for Figure 7E

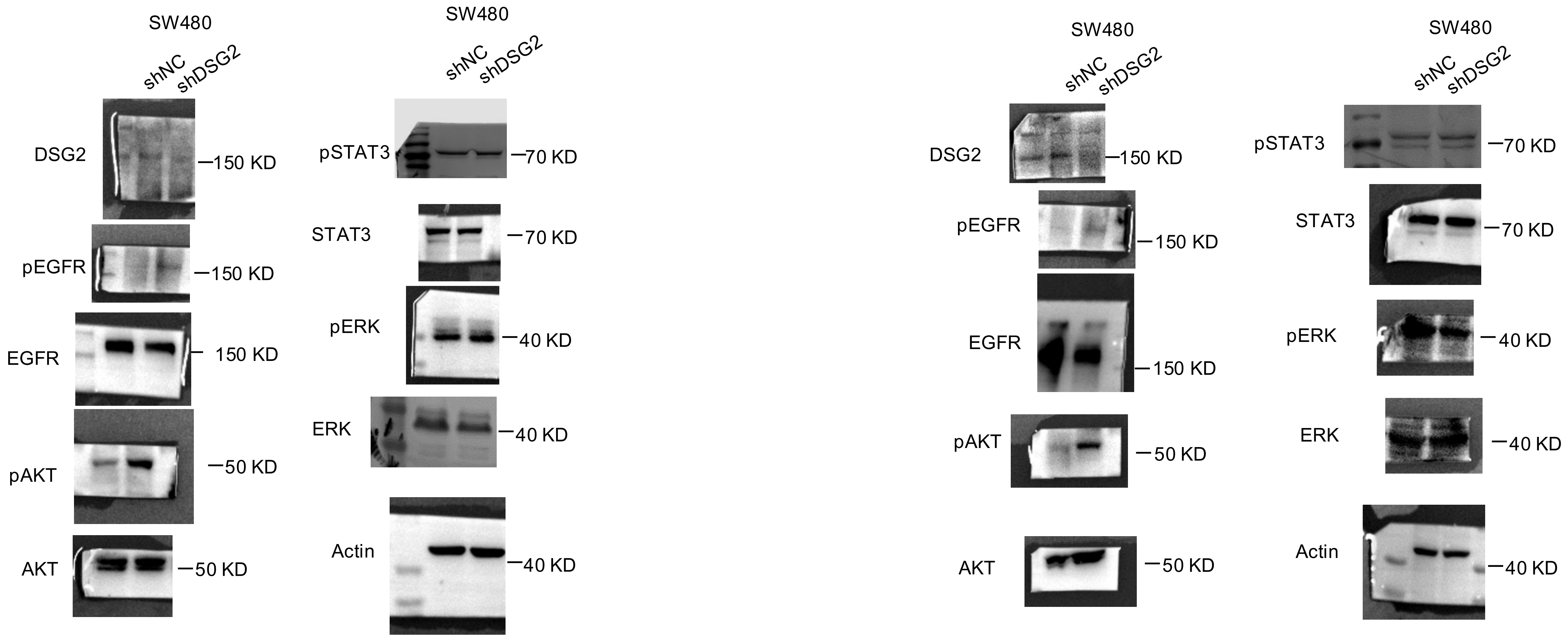

# Full unedited gel for Figure 7H

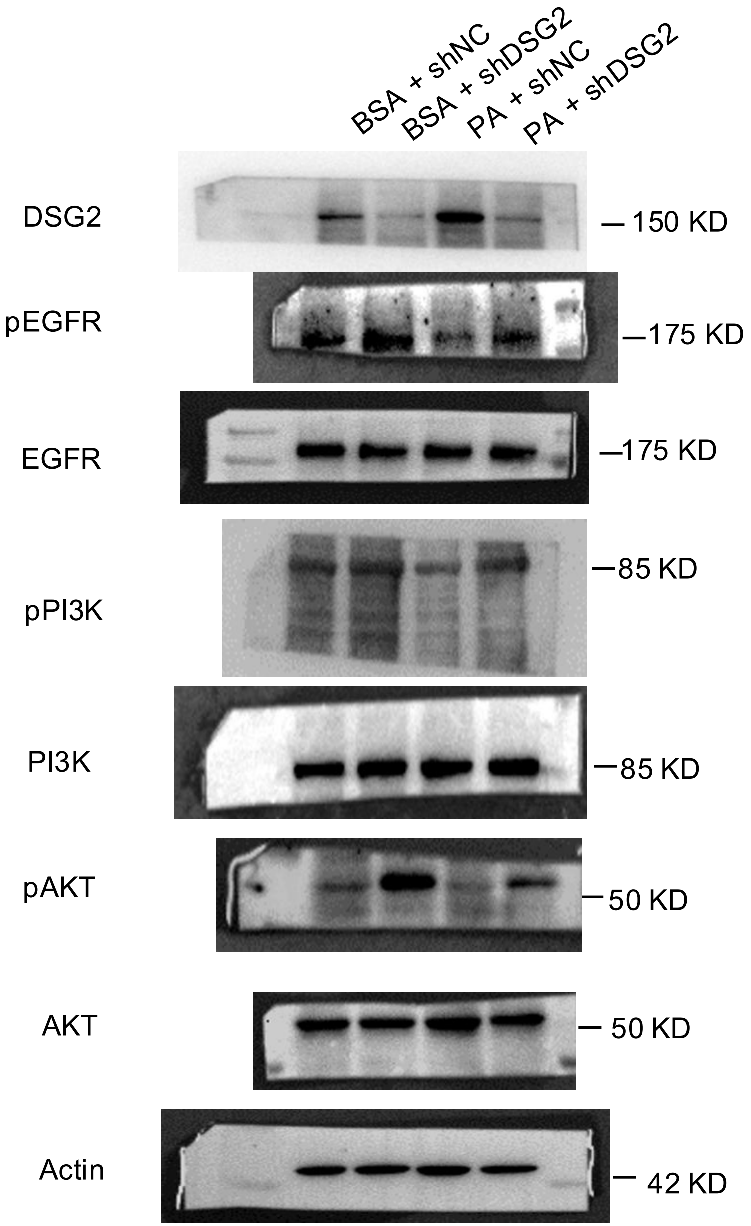

# Full unedited gel for Figure 7H

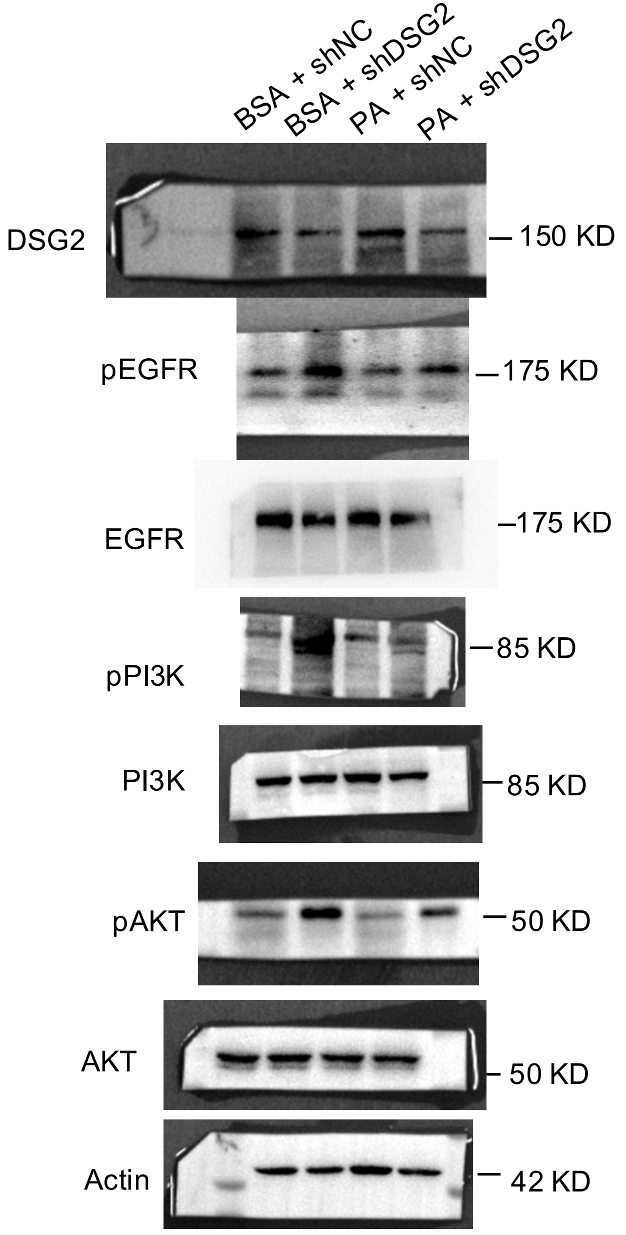


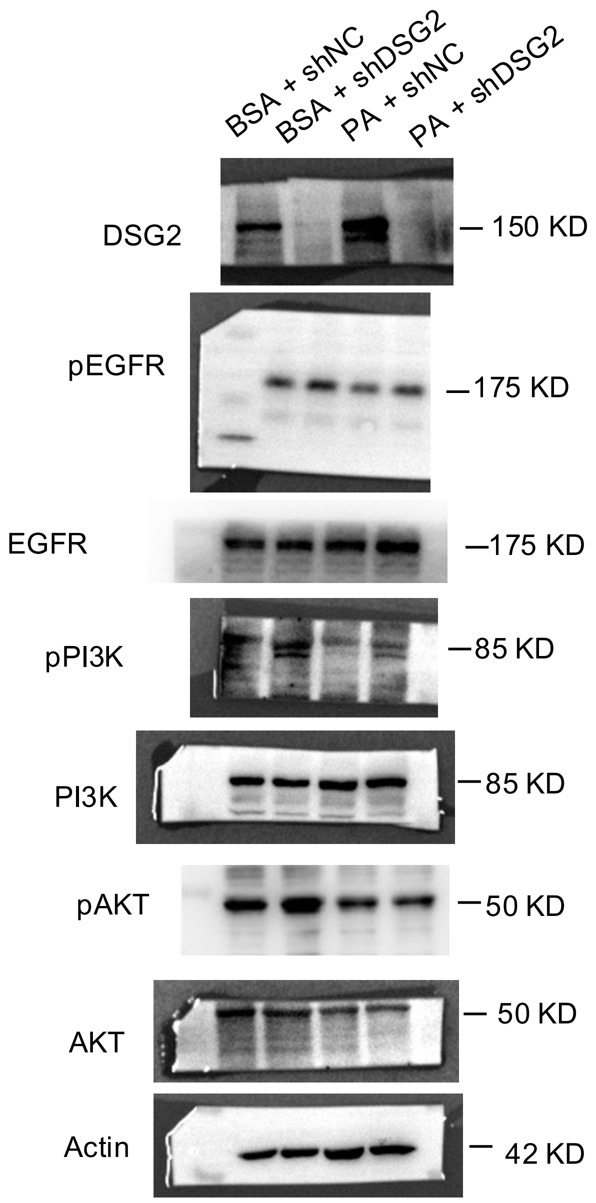

Supplement: Unedited blot and gel images [file jciinsight-11-179533-s009.pdf]
